# Evaluating Viral Pollution in Wastewater and Mediterranean Ecosystems

**DOI:** 10.1007/s12560-026-09693-3

**Published:** 2026-04-29

**Authors:** Pablo Puchades-Colera, Inés Girón-Guzmán, Enric Cuevas-Ferrando, Azahara Díaz-Reolid, Irene Falcó, Rosa Aznar, Marinella Farré, Marta Llorca, Alba Pérez-Cataluña, Gloria Sánchez

**Affiliations:** 1 VISAFELab, Department of Preservation and Food Safety Technologies, Institute of Agrochemistry and Food Technology, IATA-CSIC, Av. Agustín Escardino 7, 46980 Paterna, Valencia Spain; 2 Department of Microbiology and Ecology and Spanish Type Culture Collection (CECT), University of Valencia, MIRRI-ES, Valencia, Spain; 3 Institute of Environmental Assessment and Water Research, C/Jordi Girona, 18-26, 08034 Barcelona, Spain

**Keywords:** Mediterranean ecosystems, Human enteric viruses, Respiratory viruses, Viral faecal indicators, Surface water, Wastewater

## Abstract

Increasing water scarcity and climate-related extremes are amplifying the need for safe water reuse, however, wastewater treatment plants (WWTPs) may not fully remove viral pathogens, posing potential risks to freshwater environments. This study assessed human enteric and respiratory viruses using (RT)-qPCR, along with faecal indicators using culture-based methods, at WWTPs in two Mediterranean protected areas (Albufera Natural Park and the Ebro River region) across three campaigns. A total of influent (*n* = 33), reclaimed wastewater (*n* = 33), biosolids (*n* = 32), surface water (*n* = 33), and sediment (*n* = 29) samples were analysed. Influent wastewater contained a wide range of viral and faecal indicators, with biosolids showing similar levels. Although reclaimed water demonstrated reductions in *Escherichia coli* and somatic coliphages, these reductions remained below the ≥ 5 and ≥ 6 Log_10_ threshold required under EU water reuse regulations, respectively. In surface waters, human enteric viruses were detected at low concentrations, with human norovirus genogroup II (HuNoV GII) showing the highest incidence (36.36%). SARS-CoV-2 was detected in 15.15% of samples, while influenza A virus (IAV) was found in only one sample. Sediments yielded viral signals in 10–35% of samples, whereas *E. coli* and somatic coliphages were detected in 6.9% and 10.3% of samples, respectively. Hepatitis E virus (HEV) genotype 3f exhibited a clear winter trend, whereas faecal indicators were consistently detected across matrices. In contrast, *E. coli* and somatic coliphages showed no correlation with viral detections. As the first comprehensive survey of viral presence in these Mediterranean systems, this study provides a baseline for monitoring and managing viral contamination risks during and after extreme climate events, which may exacerbate pathogen mobilization and dissemination. Additionally, the detection of zoonotic viruses, including IAV and HEV, highlights the value of wastewater and environmental surveillance (WES) in biodiversity-rich environments.

**Supplementary Information:**

The online version contains supplementary material available at 10.1007/s12560-026-09693-3.

## Introduction

Water scarcity, driven by climate change, drought, and increasing demand, is a growing global concern that underscores the urgent need for sustainable water management, including the reuse of treated wastewater (Christou et al., 2024). Reclaimed water and sludge are increasingly reused in agriculture worldwide (Corpuz et al., 2020); however, a significant portion is still informally or inadequately treated, posing serious public health and environmental risks (Li et al., 2021). Effective treatment and management of these resources not only contribute to water resilience and food production but also support the development of a circular economy (Regitano et al., 2022).

Despite advances in treatment technologies, water reuse entails risks related to both chemical and microbiological safety. Human enteric viruses, and to a lesser extent respiratory virus, have been frequently detected in treated wastewater (Haramoto et al., 2018; Viviani et al., 2025; Yan et al., 2024), yet the extent and dynamics of viral contamination in environmental waters remain underexplored (Haramoto et al., 2018; Sims & Kasprzyk-Hordern, 2020). The discharge of untreated or insufficiently treated wastewater into natural ecosystems facilitates the introduction of these viruses into freshwater environments, where they can persist, spread, and potentially re-enter the human population through water or food chains (Hassard et al., 2017; Singh, 2021; Omatola & Olaniran, 2022).

Human enteric viruses are of particular concern due to their resilience and ability to remain infectious in aquatic environments for extended periods (Ekundayo et al., 2021; Shaffer et al., 2022; Girón-Guzmán et al., 2025). Their widespread detection in sewage and environmental matrices around the world highlights the need for enhanced monitoring and a more comprehensive understanding of their behaviour and fate in water systems. While some studies in Spain have monitored viral faecal indicators and pathogens in rivers such as the Besòs and Llobregat (Sano et al., 2010; Pérez-Sautu et al., 2012; Rusiñol et al., 2015; Jurado et al., 2019; Forés et al., 2022; Mejías-Molina et al., 2024; Toribio-Avedillo et al., 2025), ecologically sensitive and economically important regions such as the Ebro River region and the Albufera Natural Park remain largely unexplored.

This knowledge gap becomes particularly critical in the context of extreme weather events, such as the DANA (Isolated Depression at High Levels) that occurred in October 2024 in Valencia, Spain, which severely impacted the Albufera Natural Park (Mas-Coma et al., 2025). Sudden overflows during the event strongly affected wastewater treatment plants (WWTPs) and sewage networks, leading to increased faecal contamination in nearby ecosystems.

Understanding baseline levels of viral pathogens in these environments is essential not only for regular surveillance but also for assessing and mitigating risks during and after extreme events. This baseline is relevant not only to the two studied ecosystems, which are subject to urban pollution pressures, but also to other similar ecosystems facing comparable environmental challenges.

In this study, we investigate the abundance and distribution of human enteric viruses, respiratory viruses and faecal indicators across WWTPs and adjacent freshwater ecosystems (lagoon and river), which serve as habitats and reservoirs for wildlife, support important agricultural areas, and may be directly or indirectly impacted by reclaimed water from the studied WWTPs. The findings aim to provide critical baseline data for future risk assessments and to inform the development of intervention strategies, particularly in the context of climate-induced extreme weather scenarios.

## Materials and Methods

### Environmental Sample Collection

This study assessed two environmentally sensitive areas in the Mediterranean region, the Albufera Natural Park and the Ebro River region (Suppl. Table S1 and S2). The Albufera Natural Park (20,000 ha), supported by a 160,000-ha watershed, includes a lake, rice fields, marshes, the Devesa forest, and surrounding dunes. Some analysed WWTPs discharge directly into the Albufera lagoon, impacting water quality. Similarly, the Ebro Delta Natural Park, a protected Ramsar wetland since 1984, features lagoons, marshes, and bays, but is affected by agriculture and urban development. WWTP discharges into the Ebro River ultimately reach the Delta, contributing to faecal contamination and posing risks to both ecosystem health and water quality (Peris et al., 2022; Dulsat-Masvidal et al., 2023; Castaño-Ortiz et al., 2024; Garcia-Torné et al., 2025).

Sampling was conducted during three campaigns, January 2022 (winter), July 2022 (summer), and March 2023 (rainy season). A total of 66 grab water samples were collected in sterile 1 L high-density polyethylene (HDPE) containers (Labbox Labware, Spain) from influents and reclaimed waters at eleven WWTPs, five situated in or close to the Albufera Natural Park (*n* = 30) (WWTP P1-P5) and six along the Ebro River region (*n* = 36) (WWTP P6-P11), together with 32 biosolid samples (*n* = 15 and 17, respectively). All samples were obtained by plant operators. Characteristics and wastewater treatments for each WWTP analysed are summarised in Suppl. Table S3 and S4.

Additionally, surface water samples (*n* = 33) were collected approximately 1–6 km downstream of WWTPs discharge points, including 18 samples from Albufera Natural Park (SW1-6) and 15 from the Ebro River region (SW7-11). River basin sediments (*n* = 29); 17 from Albufera Natural Park (SD1-6) and 12 from Ebro River region (SD7-10) were also analysed.

Surface water grab samples were collected by directly immersing a 5 L bottle at the designated sampling points (rivers or basins). In surface waters, physico-chemical parameters (temperature (ºC), conductivity (µS/cm**)**, oxygen demand (mg/L) and pH) were measured using YSI™ Professional Plus Multiparameter Meter (Thermo Fisher Scientific, USA). Detailed information on sampling locations and physico-chemical parameters is provided in Suppl. Table S1 and S2. All samples were transported on ice to the laboratory and stored at 4 °C.

### Somatic Coliphages and *Escherichia coli* Enumeration

Somatic coliphages were quantified from 1 mL of influent wastewater and reclaimed water samples filtered through 0.45 μm pore filters, with the ‘Bluephage Easy Kit for Enumeration of Somatic Coliphages’ (Bluephage S.L., Spain), as recommended by the manufacturer. From surface waters, somatic coliphages were analysed from 1 mL of water concentrate. The detection limit (LoD) for somatic coliphages counts in influent wastewater and reclaimed water was 3.00 Log_10_ plaque-forming units (pfu)/L, while in surface water was 0.34 Log_10_ pfu/L.

For influent wastewater samples, *E. coli* enumeration was performed using the selective media Chromocult coliform agar (Merck, Darmstadt, Germany) as previously described (Garcia-Torné et al., 2025). Briefly, samples were serially diluted, and 100 µL aliquots of each dilution were spread-plated. Reclaimed water and surface water (200 mL) were quantified by filtering using a filtration manifold equipped with 0.45 μm cellulose nitrate membrane filters (Sartorius, Madrid, Spain). After incubation at 37 °C for 24 h, dark blue-violet colonies were considered positive for *E. coli*. The LoD for *E. coli* in influent wastewater was 3.00 Log_10_ colony-forming units (cfu)/L, while in reclaimed water and surface water was 1.00 Log_10_ cfu/L.

For biosolid and sediments samples, 1 g of each sample was resuspended in 10 and 100 mL of PBS for the enumeration of both somatic coliphages and *E. coli*, being the LoD 1.00 Log_10_ pfu/L and 2.00 Log_10_ cfu/L, respectively.

### Virus Concentration Methods

Water samples were artificially inoculated with 100 µL of porcine epidemic diarrhoea virus (PEDV) strain CV777 (kindly provided by Prof. Carvajal from University of Leon) at 10^8^ genome copies (gc)/mL as a coronavirus surrogate, and mengovirus (MgV) vMC_0_ (CECT 100000) at 10^7^ gc/mL as a non-enveloped virus model for recovery efficiency assessment.

Influent wastewater and reclaimed water samples (200 mL) were concentrated using the aluminium-based adsorption-precipitation method, a validated approach widely used for detecting viruses, as well as antibiotic resistance, and protozoan parasites in wastewater (AAVV, 2018; Girón-Guzmán et al., 2024; Puchades-Colera et al., 2025). The resulting pellet was resuspended in phosphate-buffered saline (PBS), yielding a final concentrate volume of approximately 2 mL. Surface water samples (5 L) were concentrated using Dead-End Hollow Fiber Ultrafiltration (DEUF) with single-use Rexeed-25 A dialysis filters (Asahi Kasei Medica Co., Ltd., Germany), followed by secondary concentration adding to the samples 50 mL of 5x polyethylene glycol solution (PEG) and placed in an orbital shaker overnight at 4 °C, as described by Cuevas-Ferrando et al. (2020). The pellet was subsequently resuspended in PBS, yielding a final volume of approximately 2.5 mL. Virus concentrates were stored at −80 °C.

For biosolid (dry weight) and sediment (wet weight) samples, 100 mg of each was resuspended in 900 µL of PBS. After vortexing, 300 µL aliquots were used for nucleic acid extraction.

### Nucleic Acid Extraction and Virus Quantification

Nucleic acid extraction from water concentrates (300 µL), as well as from biosolids and sediment suspensions (300 µL), was performed using the Maxwell^®^ RSC Instrument (Promega, Madison, USA) in combination with the Maxwell RSC Pure Food GMO and authentication kit, following the “Maxwell RSC Viral Total Nucleic Acid” program (Pérez-Cataluña et al., 2021; Girón-Guzmán et al., 2023). Nucleic acids were eluted in 200 µL of the elution buffer supplied with the commercial kit.

Viral detection of process control viruses, PEDV and MgV, was performed by reverse transcription qPCR (RT-qPCR) using the One Step PrimeScript™ RT-PCR Kit (Perfect Real Time, Takara Bio Inc., USA). Detection of norovirus (HuNoV) genogroups I and II, human astrovirus (HAstV), rotavirus (RV), hepatitis A virus (HAV), and hepatitis E virus (HEV) were carried out using the RNA UltraSense One-Step kit (Invitrogen, USA). Occurrence of crAssphage was assessed using the qPCR Premix Ex Taq™ kit (Takara Bio Inc). Pepper mild mottle virus (PMMoV) was quantified with the ‘PMMoV Faecal Indicator RT-qPCR Kit’ (Promega), according to the manufacturer’s recommendations. Detection of SARS-CoV-2 was performed using the One Step PrimeScript™ RT-PCR Kit (Perfect Real Time, Takara Bio Inc.) and targeting the N1 region of the nucleocapsid gene (CDC, 2020). The latter kit was also used for the detection of influenza A virus (IAV) and respiratory syncytial virus (RSV). Primers, probes, and PCR conditions are described in Suppl. Table S5.

Different controls were included in all assays to ensure accuracy and reliability. These consisted of a whole process control to assess sample processing efficiency (samples spiked with PEDV at 10^8^ gc/mL and MgV at 10^7^ gc/mL), a negative extraction control (PBS), and RT-qPCR controls, including a positive control (reference materials) and a negative control (RNase-free water).

Standard curves for quantification were generated using gBlock synthetic gene fragments (Integrated DNA Technologies, Inc., USA) for HuNoV GI and GII, HAstV, RV, HAV, HEV, and crAssphage. For quantification of IAV and RSV, Twist Synthetic InfluenzaV H1N1 RNA control (Twist Bioscience, USA) and purified RSV RNA (Vircell, S.L., Spain) were used, respectively. Additionally, the PMMoV Faecal Indicator RT-qPCR Kit (Promega) supplied PMMoV RNA for standard curve generation. Standard curves generated with the gBlock comprised at least nine points, each analysed in triplicate, using serial dilutions generally ranging from 10^− 2^ to 10^− 10^. Standard curves prepared with Twist Synthetic RNA and Vircell purified RNA also included a minimum of nine points (including intermediate concentrations), each run in triplicate, with serial dilutions ranging from undiluted to 10^− 4^. For PMMoV standard curve generation, seven points were included, each tested in triplicate, with serial RNA dilutions ranging from undiluted to 10^− 6^.

### HEV Sequencing

Given the highly variable occurrence of HEV in the water cycle (Fenaux et al., 2019) and the unexpectedly high detection prevalence observed during the winter sampling campaign, samples positive for HEV by RT-qPCR were further confirmed using a nested RT-PCR targeting the ORF2 region, yielding a 566 bp amplicon (Boxman et al., 2017). For cDNA synthesis, 5 µL of RNA was reverse transcribed in a final volume of 20 µL using SuperScript™ III Reverse Transcriptase (50 U/µL) (Thermo Fisher Scientific, USA). The reaction mix included 5× Expand RT Buffer, a 25 mM dNTP mix, 100 µM HEV-orf2-ro-ch reverse primer (Suppl. Table S6), 100 mM DTT, and nuclease-free water. The reverse transcription protocol consisted of incubation at 65 °C for 5 min followed by 10 min at 50 °C and an inactivation step at 80 °C for 10 min. The first-round PCR amplification was performed in a 50 µL reaction mixture containing 10 µL of cDNA, 1× Q5^®^ PCR Buffer (New England BioLabs, USA), 2.5 mM of each dNTP, 10 µM HEV-orf2-fo-ch forward primer (Suppl. Table S6), and Q5^®^ Hot Start High-Fidelity DNA Polymerase (5 U/µL) (New England BioLabs). The thermal cycling program included an initial denaturation at 95 °C for 5 min, followed by 35 cycles of denaturation at 95 °C for 30 s, annealing at 42 °C for 30 s, and extension at 60 °C for 45 s. Reactions were carried out on a SimpliAmp™ Thermal Cycler (Thermo Fisher Scientific).

Nested PCR was performed to amplify a 566 bp fragment of the HEV ORF2 region from the first-round PCR product. The reaction was carried out in a final volume of 50 µL, containing 1 µL of the first PCR product, 1× Q5^®^ PCR Buffer (New England BioLabs), 2.5 mM of each dNTP, 10 µM each of forward (HEV-orf2-fi-ch) and reverse (HEV-orf2-ri-ch) primers, and Q5^®^ Hot Start High-Fidelity DNA Polymerase (5 U/µL) (New England BioLabs). The thermal cycling conditions included an initial denaturation at 95 °C for 5 min, followed by 40 cycles of 30 s at 95 °C, 30 s at 60 °C, and 20 s at 72 °C. All reactions were performed on a SimpliAmp™ Thermal Cycler (Thermo Fisher Scientific).

PCR products (10 µL) were visualized by electrophoresis on a 1.5% agarose gel prepared in 1× TBE buffer and purified using the GFX PCR DNA and Gel Band Purification Kit (Cytiva, USA) following manufacturer instructions. The purified amplicons were then submitted for automated Sanger sequencing at the Central Service for Experimental Research (SCSIE) core facilities from the University of Valencia. Resulting sequences were genotyped using the RIVM HEV Typing Tool (https://www.rivm.nl/mpf/typingtool/hev/). Sequences were deposited at GeneBank under the accession numbers PX417640, PX417641, and PX417642.

### Statistical Analysis

All statistical analyses were performed using RStudio version (2023.04.21) running R version 4.3.0, with statistical significance set at *p*-value < 0.05. The data was tested for normality distribution using the Shapiro-Wilk test with a 95% confidence interval. The assumption of homogeneity of variances was assessed using Levene’s test to verify the equality of variances across groups. Temporary trends in human enteric viruses (HuNoV GI and GII, RV, HAstV, and HEV) among sampling campaigns (winter, summer and rainy) were evaluated by Kruskal-Wallis test with post-hoc Dunn comparisons with Bonferroni adjusted correction test and One-Way ANOVA followed by Tukey’s Honest Significant Difference (HSD) test for post hoc multiple comparisons. In addition, differences in the mean removal efficiencies (difference between influent wastewater and reclaimed water levels) of human enteric viruses, respiratory viruses, and faecal indicators among WWTPs were assessed using the same statistical approach. For samples testing negative for the different targets (< LoD), the theoretical LoD of the method was used for the calculation of Log reductions. Spearman’s correlation matrix of human enteric viruses, viral faecal indicators and physico-chemical properties of surface water was performed using package ‘ggstatsplot’ described by (Patil, 2021), and p-values settled at 0.05 were adjusted by Holm-Bonferroni method. For Spearman correlation analysis, negative results for the different targets were assigned a value of 0.

## Results and Discussion

### Tracking Viral Contaminants and Faecal Indicators in WWTPs Surrounding the Albufera Natural Park and Ebro River Region

**Fig. 1:**
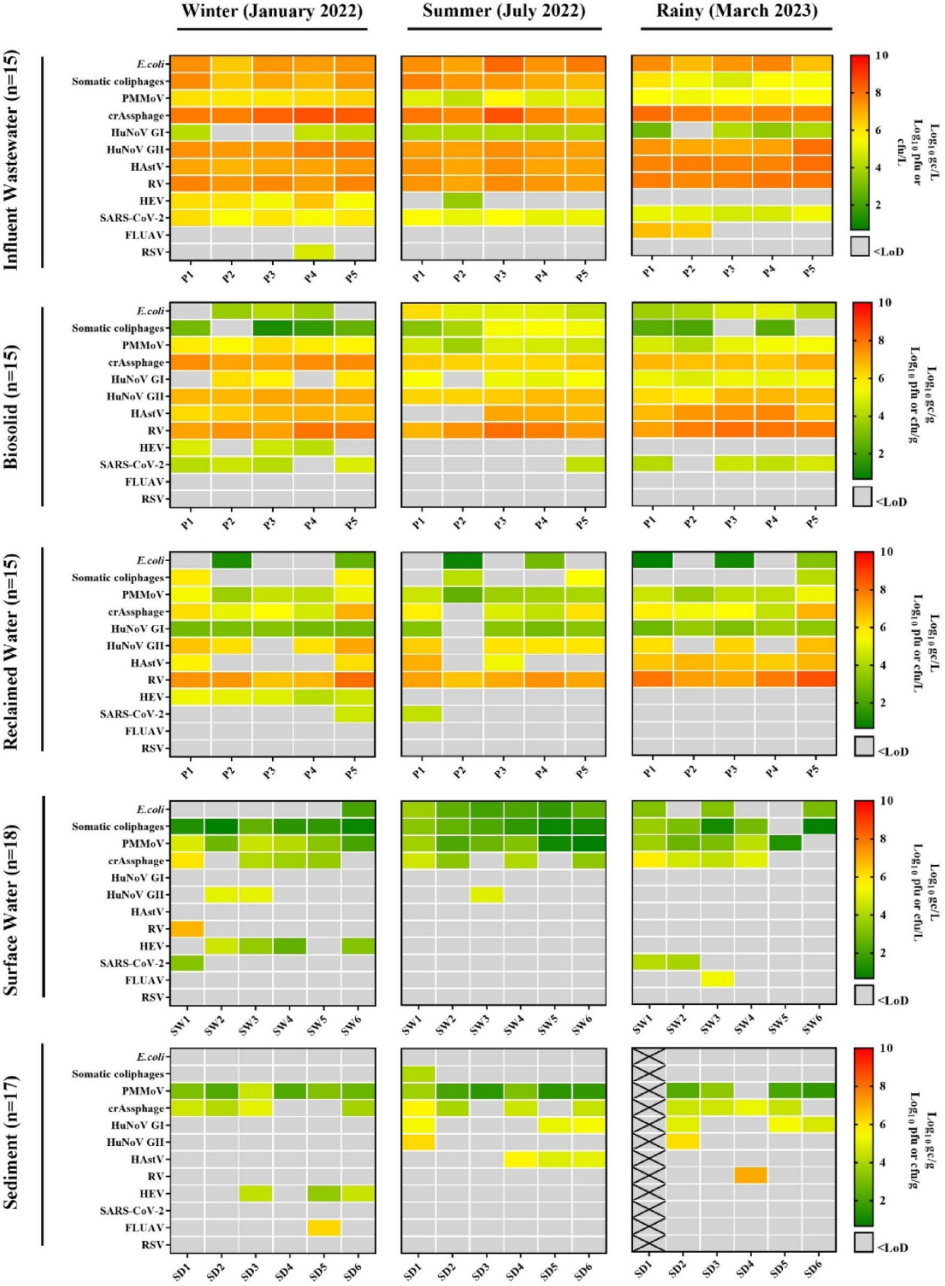
Levels of faecal indicators, human enteric viruses (HuNoV GI and GII, RV, HAstV, and HEV) and human respiratory viruses along influent wastewater, biosolids, reclaimed water, surface water and sediments in the Albufera Natural Park collected in winter (January 2022), summer (July 2022) and the rainy season (March 2023). P1-P5 denote the five different WWTPs analysed in Albufera Natural Park area, while SW1-SW6 and SD1-SD6 refer to surface water and sediment samples, respectively. PMMoV, pepper mild mottle virus; HuNoV GI, human norovirus genogroup I; HuNoV GII, human norovirus genogroup II; HAstV, human astrovirus; RV, rotavirus; HEV, hepatitis E virus; SARS-CoV-2, severe acute respiratory syndrome coronavirus 2; IAV, influenza A virus; RSV, respiratory syncytial virus; LoD, limit of detection; gc, genome copies; pfu, plaque-forming units; cfu, colony-forming units; WWTP, wastewater treatment plant. Black crosses mean unavailable samples

**Fig. 2:**
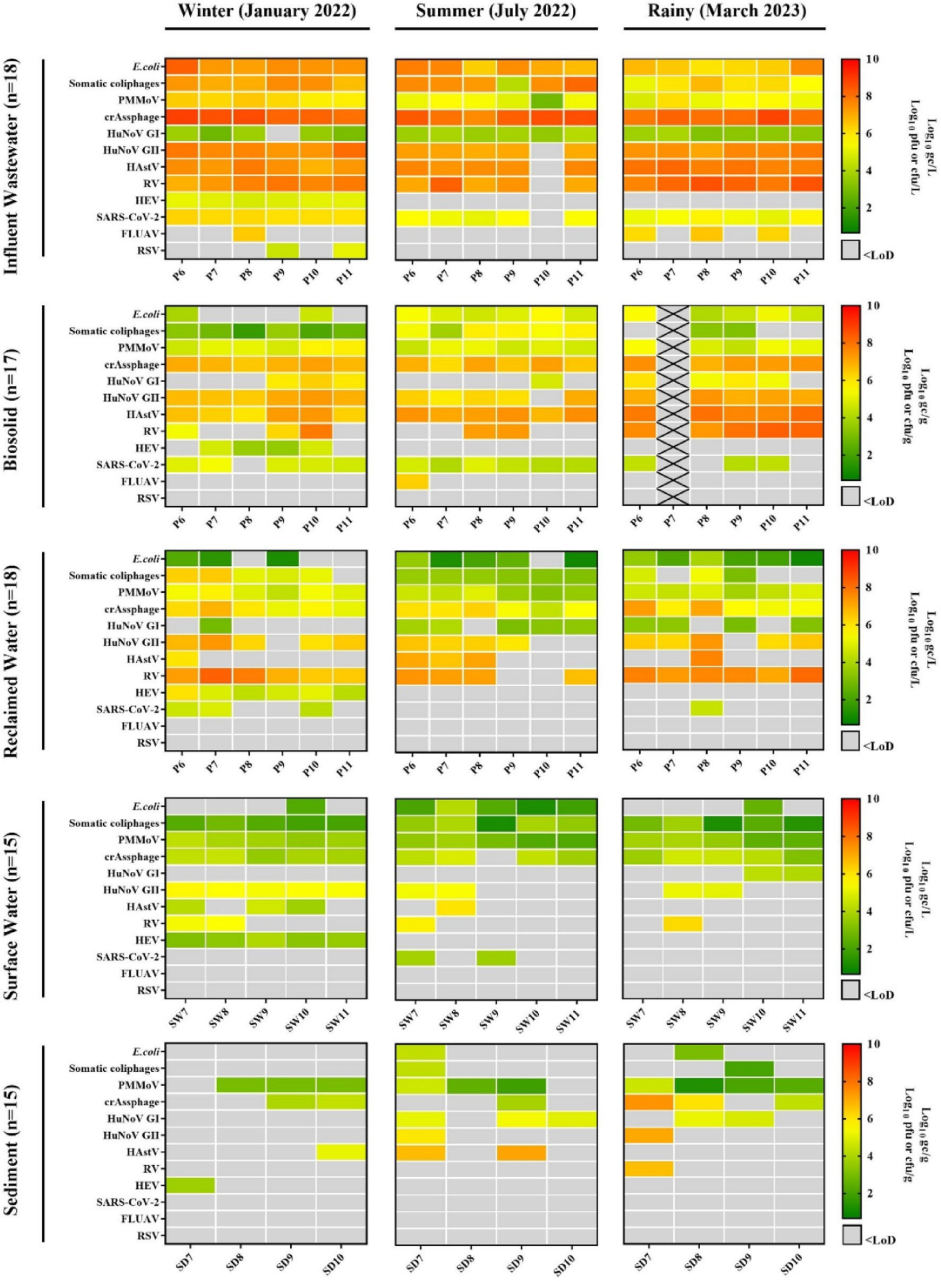
Levels of faecal indicators, human enteric viruses (HuNoV GI and GII, RV, HAstV, and HEV) and human respiratory viruses along influent wastewater, biosolids, reclaimed water, surface water and sediments in Ebro River region collected in winter (January 2022), summer (July 2022) and the rainy season (March 2023). P6-P11 denote the six different WWTP analysed in the Ebro River region, while SW7-SW11 and SD7-SD10 refer to surface water and sediment samples, respectively. PMMoV, pepper mild mottle virus; HuNoV GI, human norovirus genogroup I; HuNoV GII, human norovirus genogroup II; HAstV, human astrovirus; RV, rotavirus; HEV, hepatitis E virus; SARS-CoV-2, severe acute respiratory syndrome coronavirus 2; IAV, influenza A virus; RSV, respiratory syncytial virus; LoD, limit of detection; gc, genome copies; pfu, plaqu-forming units; cfu, colony-forming units; WWTP, wastewater treatment plant. Black crosses mean unavailable samples

In this study, a total of 66 wastewater samples were analysed, comprising 33 influent wastewater samples and 33 reclaimed water samples. Of these, 30 samples (15 influent wastewater and 15 reclaimed water) were collected around the Albufera Natural Park, while 36 samples (18 influent wastewater and 18 reclaimed water) were obtained from the Ebro River region over a one-year period. Samples were analysed by (RT)-qPCR for the presence of human enteric and respiratory viruses, including HuNoV GI and GII, HAstV, HAV, HEV, RV, SARS-CoV-2, IAV and RSV. Additionally, viral faecal indicators, crAssphage and PMMoV, along with somatic coliphages and total *E. coli* (both quantified by plate count), were assessed (Figs. 1 and 2 and Suppl. Table S7 and Suppl. Table S8).

As expected, the concentration (Figs. 1 and 2) and prevalence (Suppl. Fig. S1 and S2) of some human enteric viruses (HuNoV GI and GII, RV, HAstV) in influent wastewater samples were high, consistent with previous studies (Haramoto et al., 2018; Corpuz et al., 2020; Yan et al., 2024), with values ranging from 2.80 to 8.57 Log_10_ gc/L. In both analysed areas, RV was detected at the highest concentrations (6.91–8.57 Log_10_ gc/L), followed by HAstV (6.84–8.18 Log_10_ gc/L), HuNoV GII (6.97–8.09 Log_10_ gc/L) and HuNoV GI (2.80–4.43 Log_10_ gc/L) (Suppl. Table S7 and 8). This trend is consistent with observations by Santiso-Bellón et al. (2020) and Carmona-Vicente et al. (2024) in influent wastewater from the Valencian region, where higher levels of RV were also reported. Interestingly, HEV, primarily detected during the winter campaign (January 2022), was found in both areas at concentrations ranging from 4.57 to 6.54 Log_10_ gc/L. Furthermore, two samples from the winter campaign (from the P3 and P4 WWTPs) were sequenced by Sanger, corresponding to genotype 3f (Acc. numbers PX417641 and PX417642). The presence of HAV was also investigated but was not detected in any samples from both areas, consistent with recent surveys of WWTPs in the region reporting HAV prevalence of only 3.7% and 6%, with concentrations ranging from 3.05 to 5.46 Log_10_ gc/L (Randazzo et al. 2019; Girón-Guzmán et al. 2024a, 2024b). Interestingly, no temporal trends were observed for HuNoV GI and GII, RV and HAstV, and results were similar across WWTPs, suggesting comparable patterns of viral faecal contamination in both areas.

Regarding respiratory viruses (Figs. 1 and 2), SARS-CoV-2 was consistently detected in all influent wastewater samples, with mean concentrations of 5.36–5.67 Log_10_ gc/L (Pérez-Cataluña et al. 2021; Sanjuán and Domingo-Calap 2021; Girón-Guzmán et al. 2024a, 2024b; Toribio-Avedillo et al. 2023), and was more prevalent than other respiratory viruses (Suppl. Fig. S1 and S2). In contrast, IAV and RSV were detected in only 9 of the 33 samples, at concentrations ranging from 6.14 to 6.68 Log_10_ gc/L (6/33) for IAV and from 4.48 to 5.08 Log_10_ gc/L (3/33) for RSV. In both studied areas, RSV was detected primarily during the winter sampling campaign, whereas IAV was detected mainly during the rainy season in the Albufera Natural Park WWTPs and during both the rainy and winter seasons in the Ebro River region WWTPs. These findings are consistent with those reported by Girón-Guzmán et al. (2024a, 2024b) and Toribio-Avedillo et al. (2023), who observed a seasonal pattern for both respiratory viruses, mainly concentrated in the winter or late winter months.

Faecal indicators were detected in all influent wastewater samples (*n* = 33). CrAssphage exhibited the highest concentrations (7.31–8.85 Log_10_ gc/L), followed by PMMoV (2.83–6.43 Log_10_ gc/L). Somatic coliphages were present at 4.19–8.16 Log_10_ pfu/L, and *E. coli* counts averaged 6.00–8.29.00.29 Log_10_ cfu/L. These results are consistent with previously reported global crAssphage levels, which range from 4.8 to 12.0 Log_10_ gc/L (Ahmed et al., 2018; Farkas et al., 2019; Malla et al., 2019; Sabar et al., 2022), and align specifically with values reported in high-income countries, including Spain (7.4–10.0 Log_10_ gc/L) (García-Aljaro et al., 2017). Furthermore, the concentrations observed for somatic coliphages, with 100% prevalence, were comparable to those reported in previous studies (Lucena et al., 2004; Truchado et al., 2021a; Girón-Guzmán et al. 2024b; Puchades-Colera et al. 2024).

The reuse of treated wastewater and biosolids is increasingly necessary to address water scarcity and meet agricultural and environmental needs. However, these practices raise public health concerns due to the presence of viral pathogens that may not be completely removed by WWTPs. To mitigate these risks, the European Union implemented Regulation (EC) 2020/741 (European Union, 2020), effective June 2023, establishing minimum quality requirements for reclaimed water used in agricultural irrigation and other applications, including performance targets for the removal of *E. coli* and human enteric viruses with mandatory validation monitoring to ensure compliance.

In this study, RV was the most prevalent virus in reclaimed water samples from both areas (*n* = 33), (Figs. 1 and 2 and Suppl. Table S9 and S10), being detected in 31 of 33 samples at an average concentration of 7.32 ± 0.54 Log_10_ gc/L. HuNoV GI and GII were detected in 24 and 25 of 33 samples, respectively, with average concentrations of 3.23 ± 0.32 Log_10_ gc/L and 6.40 ± 0.40 Log_10_ gc/L. HAstV was less prevalent, found in 14 out of 33 samples at an average of 6.56 ± 0.62 Log_10_ gc/L. Our levels of HuNoV GII and HAstV were comparable to those reported by Girón-Guzmán et al. (2024b), while slightly lower levels were observed for HuNoV GI and RV. These minor differences (approximately 0.1–0.3 Log_10_ gc/L) may be attributed to variations between WWTPs and the fact that the Girón-Guzmán et al. (2024b) study encompassed a full year of sampling. HEV was detected in all samples from the winter campaign in both areas, with an average concentration of 4.83 ± 0.53 Log_10_ gc/L (11/33), contrasting with other studies that reported its absence or low detection in reclaimed water (Clemente-Casares et al. 2003; Rusiñol et al. 2015; Cuevas-Ferrando et al. 2020; Girón-Guzmán et al. 2024b). HAV was not detected in any of the reclaimed water samples.

Regarding respiratory viruses, SARS-CoV-2 was detected in only six reclaimed water samples (P1, P5, P6, P7, P8 and P10), with an average concentration of 4.51 ± 0.22 Log_10_ gc/L, consistent with previous reports (Randazzo et al., 2020; Rimoldi et al., 2020). In contrast, IAV and RSV were reduced to undetectable levels during treatment. These findings align with earlier studies (Heijnen & Medema, 2011; Williams et al., 2024) showing that sewage treatment successfully reduces the concentration of a range of pathogens, including respiratory viruses (Girón-Guzmán et al. 2024a, 2024b). However, the detection of viral RNA in these matrices does not necessarily imply a health risk and will also depend on the effectiveness of wastewater treatment, which in turn is influenced by multiple factors, such as facility-specific process specifications and the characteristics of the treated water (e.g., turbidity and suspended solids) (Guo et al., 2021; Canh et al., 2022; Foster et al., 2024).

PMMoV and crAssphage were consistently detected in all 33 and 32 of 33 reclaimed water samples, respectively. Average concentrations ranged from 4.22 to 4.54 Log_10_ gc/L for PMMoV and from 5.52 to 5.83 Log_10_ gc/L for crAssphage. Somatic coliphages were detected in 19 of 33 samples at 4.56 ± 1.15 Log_10_ pfu/L, while *E. coli* was found in 19 of 33 samples at 1.89 ± 1.00 Log_10_ cfu/L. These values are comparable to those reported by Girón-Guzmán et al. (2024b), who observed similar somatic coliphage levels (5.73 Log_10_ pfu/L) but higher *E. coli* concentrations (6.43 Log_10_ cfu/L). In contrast, López-Gálvez et al. (2016) reported *E. coli* in 100% of reclaimed water samples, with concentrations ranging from 3.34 to 3.91 Log_10_ cfu/L and maximum levels reaching 4.92 to 5.05 Log_10_ cfu/L. Moreover, Puchades-Colera et al. (2024) reported somatic coliphage concentrations approximately 2.00 Log_10_ pfu/L lower. Overall, our somatic coliphage and *E. coli* measurements align with values previously compiled for reclaimed water in Spain by Truchado et al. (2021b) and Kelmer et al. (2023).

Under Regulation (EC) 2020/741 (European Union, 2020) and based on the *E. coli* levels detected in this study, the reclaimed water would be classified as Category B (≤ 100 cfu/100 mL), suitable for irrigating crops consumed raw, provided the edible part grows above ground and does not come into direct contact with the reclaimed water. In addition, reclaimed water from the analysed WWTPs is discharged either directly into the Ebro River basin, into the surrounding areas of the Albufera Natural Park, or into the Mediterranean Sea (CNV, 2025). This discharge may significantly impact the water quality of both the Albufera Natural Park and the Ebro Delta, the latter being an important area for shellfish cultivation (GENCAT, 2023).

**Fig. 3:**
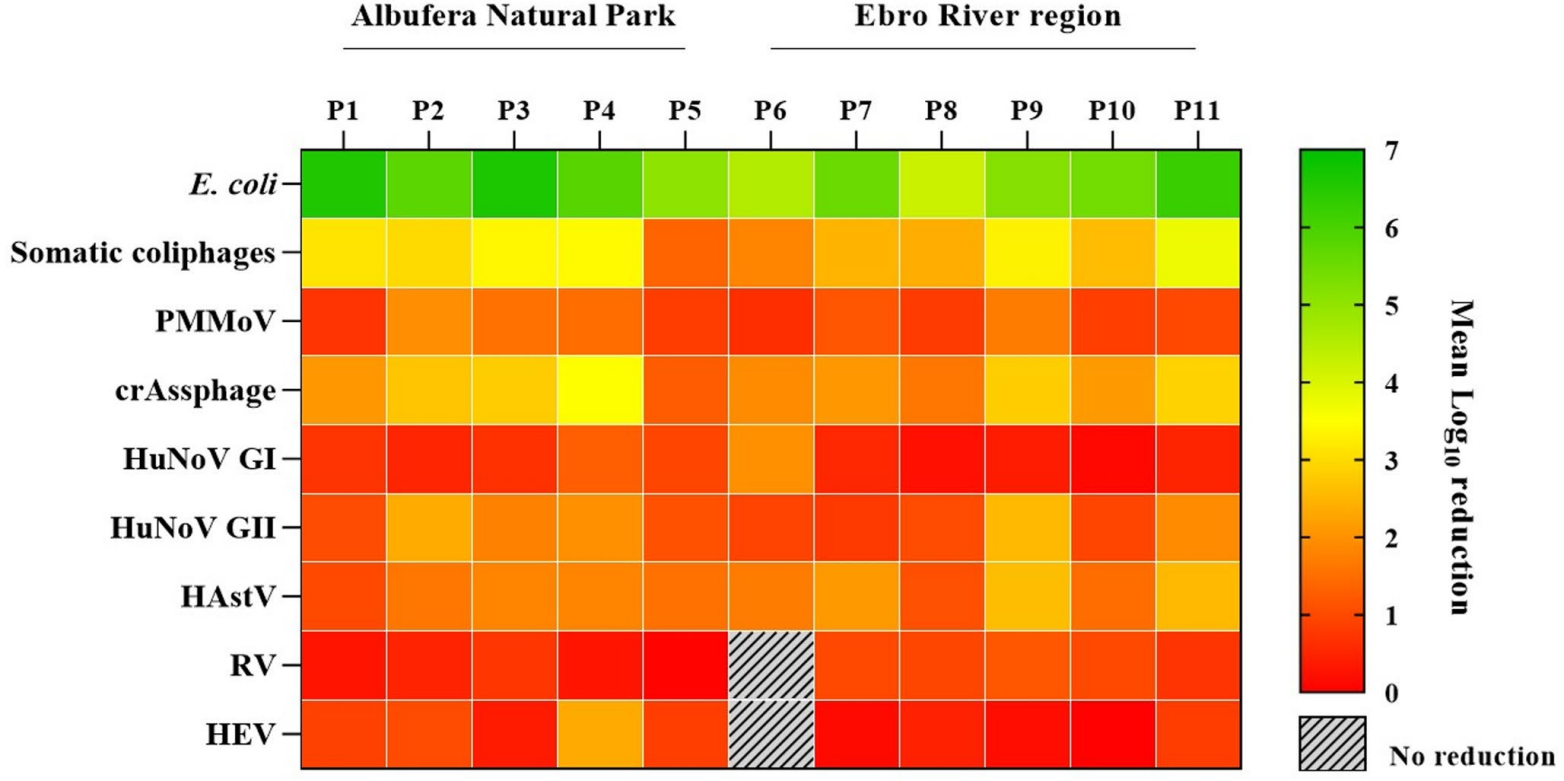
Removal efficiency of faecal indicators and human enteric viruses (Log_10_) between influent wastewater and reclaimed water collected from eleven WWTPs surrounding the Albufera Natural Park (P1-P5) and in the Ebro River region (P6-P11). PMMoV, pepper mild mottle virus; HuNoV GI, human norovirus genogroup I; HuNoV GII, human norovirus genogroup II; HAstV, human astrovirus; RV, rotavirus; HEV, hepatitis E virus; gc, genome copies; pfu, plate-forming units; cfu, colony-forming units; WWTP, wastewater treatment plant

Overall, removal of human enteric viruses (HuNoV GI and GII, RV, HAstV, HEV and HAV) ranged from 0.01 to 2.59 Log_10_, with the lowest mean reduction observed for HEV in P5 and the highest for HAstV in P9 (Fig. 3). Furthermore, HEV achieved the lowest mean reductions (0.70 ± 0.67 Log_10_), followed by RV and HuNoV GI (0.71 ± 0.45 and 0.77 ± 0.76 Log_10_). Conversely, HAstV showed the highest removal with mean reductions of 1.80 ± 0.83 Log_10_. No differences were observed in mean reductions for each human enteric virus between WWTPs, considering that all WWTPs in the Ebro River region include tertiary treatment, except P8 and P9, and that P2 and P6 are the only WWTPs in which chlorination is used as the sole tertiary treatment (Suppl. Table S3 and S4). In addition, P6 was the only WWTP where RV and HEV did not show a reduction in levels after wastewater treatment. For risk assessment purposes, it is important to note that detection was performed using RT-qPCR, which does not provide information on viral infectivity in the samples (Leifels et al., 2021; Canh et al., 2022).

Moreover, the analysed WWTPs (Suppl. Table S1 and Suppl. Table S2) were effective at removing cultivable bacterial indicators, with *E. coli* completely removed from 12 of the 33 reclaimed water samples, and mean reductions in the remaining samples ranging from 4.91 to 5.37 Log_10_. Furthermore, no significant differences in mean reductions were observed between WWTPs. Somatic coliphage reductions ranged from 0.44 to 5.01 Log_10_. Notably, somatic coliphages were completely removed from 14 of the 33 reclaimed water samples. P3 and P4 were the only WWTPs where complete removal was observed across all three sampling campaigns (Fig. 3), with mean theoretical reductions of 3.38 ± 1.40 and 3.43 ± 0.83 Log_10_, respectively. However, these values did not differ significantly from those observed in P1 (3.12 ± 1.56 Log_10_). P5 was the least effective WWTP in removing somatic coliphages (*p* = 3 × 10^− 2^). This WWTP is located in the surroundings of the Albufera Natural Park, where somatic coliphages were detected in reclaimed water across all sampling campaigns, with an average removal of only 1.36 ± 0.19 Log_10_. Similarly, P6 located in the urban area of the Ebro River region, also showed relatively low mean reductions (1.82 ± 1.91 Log_10_). Despite acceptable *E. coli* levels, the reclaimed water did not achieve the minimum ≥ 6 Log_10_ reduction of somatic coliphages required for Category A waters. PMMoV was identified as the most resistant viral indicator to wastewater treatment. Moreover, no significant differences in mean reductions were observed between WWTPs for PMMoV, with an overall mean reduction of 1.16 ± 0.60 Log_10_. Nevertheless, P2 (1.95 ± 0.32 Log_10_) and P9 (1.69 ± 0.28 Log_10_) showed the highest mean reductions.

For crAssphage, considering the overall removal across the eleven WWTPs (2.34 ± 0.89 Log_10_), P5 was the least effective (*p* = 3 × 10^− 2^), while P4 showed the highest removals (3.51 ± 0.31 Log_10_). It should be noted that these lower reductions, compared to culturable indicators, are based on (RT)-qPCR data; therefore, the removal of infectious viruses may be higher than that is indicated by genome quantification.

The use of biosolids from WWTPs as agricultural amendments can improve soil fertility but also poses a risk of pathogen transfer to crops and surrounding water bodies, particularly during rainfall events that promote surface runoff (Wong et al., 2012; Popoola et al., 2023).

In the present study, RV was the most abundant human enteric virus, detected in 25 out 32 samples at an average concentration of 7.48 ± 0.65 Log_10_ gc/g. HuNoV GII was also consistently detected (31/32) at 6.68 ± 0.40 Log_10_ gc/g, followed by HAstV, which was present in 30/32 samples at 7.07 ± 0.55 Log_10_ gc/g, and HuNoV GI, found in 20/32 samples at 5.50 ± 0.45 Log_10_ gc/g. Similar to influent wastewater and reclaimed water, HEV was only detected during the winter sampling campaign, in 7 out of 32 samples at 4.22 ± 0.54 Log_10_ gc/g (Suppl. Table S11 and 12). Among human respiratory viruses, IAV was detected in a single biosolid sample at 6.38 ± 0.00 Log_10_ gc/L. In contrast, SARS-CoV-2 was detected in 23 out of 32 samples, with an average concentration of 4.50 ± 0.35 Log_10_ gc/g, while RSV was not detected.

CrAssphage and PMMoV were detected in all biosolid samples (*n* = 32), with mean concentrations of 6.87 ± 0.41 and 5.16 ± 0.0.56 Log_10_ gc/g, respectively, in agreement with values reported by Girón-Guzmán et al. (2024b). Somatic coliphages were present in 26 of 32 samples (3.58 ± 1.48 Log_10_ pfu/g), approximately 3 Log_10_ pfu/g lower than results reported by Girón-Guzmán et al. (2024b) while *E. coli* was detected in 26 of 32 samples (4.65 ± 0.65 Log_10_ cfu/g). These concentrations are lower than those commonly reported in biosolids (~ 6.00 Log_10_ cfu/g for *E. coli* and 4.00–5.30 Log_10_ pfu/g for somatic coliphages (reviewed by Martín-Díaz et al., 2020). Generally, substantial amounts of coliphages remain in most types of biosolids, but concentrations of faecal indicators may vary substantially according to the received treatment (Martín-Díaz et al., 2020). Although enteric virus infectivity could not be directly assessed, somatic coliphages were detected using a culture-based assay, suggesting that infectious viruses may persist in biosolids.

These findings underscore the potential risk of viral transfer to the Ebro River and Ebro Delta Natural Park through the water discharge to the river and the use of reclaimed water and biosolids in agriculture. As observed in most WWTPs from the Albufera region, current treatment processes for water and biosolids intended for agricultural reuse need to be enhanced to minimize the spread of viral pathogens into aquatic ecosystems. This issue is particularly critical for food safety due to the proximity of both regions to shellfish harvesting areas and fruit and vegetable growing zones. These food products are especially susceptible to contamination from uncontrolled discharges or the use of untreated biosolids. When consumed raw or only partially cooked, they have been linked to viral foodborne outbreaks (Nemes et al., 2023; Roy et al., 2024; WHO, 2024). Strengthening treatment protocols and implementing targeted environmental surveillance could help mitigate these risks.

### Assessment of Viral and Faecal Pollution in the Albufera Natural Park Ecosystem

While the presence of human enteric viruses in surface water used for irrigation and drinking water production is well documented (López-Gálvez et al., 2016; Boehm et al., 2019; Opere et al., 2020; Takuissu et al., 2023; Yan et al., 2024), data on their occurrence in natural parks remain limited. This gap is noteworthy, as water in these environments may be used for irrigation and also represents a valuable resource for wastewater and environmental surveillance (WES). Particularly for the surveillance of emerging zoonotic viruses such as influenza A viruses, within wetland-based ecosystems and habitat of wild birds (Heijnen & Medema, 2011; Ahrens et al., 2023; Perlas et al., 2023).

Across the three sampling campaigns, HEV was the most prevalent human enteric virus detected in the Albufera surface waters (Suppl. Table S13), found in 4 of 18 samples during the colder months, with an average concentration of 3.45 ± 0.84 Log_10_ gc/L. Sanger sequencing identified the genome as belonging to clade 1, genotype 3f (acc. number PX417640), a lineage commonly detected in pigs, wild boars, and humans (Fenaux et al., 2019; Rau et al., 2024; Garbuglia et al., 2024). This finding is noteworthy, as WES monitoring can help detect infections in wildlife reservoirs and provide insights into seasonal transmission dynamics, particularly the observed association with colder periods. Even so, the presence of this genotype in the samples obtained from WWTP may indicate that the source is primarily human. HuNoV GII was detected in three samples at a concentration of 5.02 ± 0.07 Log_10_ gc/L, while RV was identified in a single sample at 6.84 ± 0.11 Log_10_ gc/L (Fig. 1 and Suppl. Table S13). Similar incidences of these viruses have been reported in urban rivers by Mackowiak et al. (2018). But, generally, lower concentration (approximately 1–2 Log_10_ gc/L) was summarised in different studies (Haramoto et al., 2018; Yan et al., 2024). By contrast, HuNoV GI and HAstV were not detected in any of the samples, despite their frequent association with sewage contamination.

SARS-CoV-2 was detected in 3 out of 18 surface water samples, with an average concentration of 3.76 ± 0.48 Log_10_ gc/L, consistent with previous reports in surface waters were concentrations ranged from 1.99 to 6.50 Log_10_ gc/L (Atoui et al., 2023). In contrast, RSV was not detected in any of the samples. IAV was found in a single sample at 5.27 ± 0.10 Log_10_ gc/L. The presence of IAV in the Albufera, where migratory birds are present, is particularly relevant, as it highlights the potential of a WES approach to track viral circulation and the introduction of IAV strains into a region.

Somatic coliphages were detected in 17 out 18 surface water samples with an average of 1.84 ± 0.97 Log_10_ pfu/L and followed by total *E. coli*, found in 10 samples at 2.56 ± 0.66 Log_10_ cfu/L. Among all viral faecal indicators detected by (RT)-qPCR, PMMoV was the most prevalent, present in 17 out of 18 samples with a mean concentration of 2.93 ± 1.22 Log_10_ gc/L, followed by crAssphage detected in 12 out of 18 samples at an average concentration of 4.37 ± 0.89 Log_10_ gc/L (Fig. 1 and Suppl. Table S13). The concentrations observed for crAssphage are consistent with previous reports (2.6–9.0 Log_10_ gc/L) (Sabar et al., 2022), whereas PMMoV levels were lower than those reported by Farkas et al. (2020), who documented higher concentrations.

Additionally, sediment samples were collected from the same locations as the surface water samples. While information on viral pathogens in surface waters is already limited in the studied ecosystems, even less is known about their presence in sediments, which may act as a reservoir for pathogens that can be mobilized during rainfall events through particulate resuspension or changes in water conditions (Hassard et al., 2016; Sassi et al., 2020; Drummond et al., 2023). Given the increasing frequency of climate-driven extreme precipitation events in the Mediterranean region, assessing the prevalence of these pathogens in sediments is particularly important (García-Aljaro et al., 2017). In the analysed samples, HuNoV GI and HAstV were not detected in the Albufera surface waters; however, HuNoV GI was detected in 6 of the 17 sediments samples (5.15 ± 0.26 Log_10_ gc/g), and HAstV was detected in 3 of the 17 samples (5.22 ± 0.42 Log_10_ gc/g) (Fig. 1 and Suppl. Table S14). RV was detected in a single sediment sample at 7.02 ± 0.02 Log_10_ gc/g. HuNoV GII was also identified in 2 of the 17 samples, with an average concentration of 6.07 ± 0.18 Log_10_ gc/g. HEV was detected only during the winter sampling campaign, in 3 of the 17 samples, with a mean concentration of 4.10 ± 0.56 Log_10_ gc/g. The absence of human enteric viruses in surface waters, contrasted with their detection in sediments, often at high concentrations, reinforces the role of sediments as key reservoirs in faecal-impacted environments. This pattern is consistent with previous findings showing that sediments efficiently accumulate and preserve human enteric viruses (Hassard et al., 2016; Cioffi et al., 2020). Our results confirm that sediments can retain viral genomes even when they are undetectable in the water column, highlighting their importance for evaluating environmental persistence and exposure risks.

IAV was found in one sediment sample from the summer campaign at 6.23 ± 0.14 Log_10_ gc/g. It is well established that viral adhesion to surfaces or materials can enhance resistance to environmental stressors (Martín-Díaz et al., 2020; Girón-Guzmán et al., 2025), and aquatic environments may act as potential reservoirs for influenza viruses (Kenmoe et al., 2024), particularly in sediments (Lang et al., 2008). Establishing an environmental baseline is therefore essential for assessing the persistence and potential infectivity of influenza A viruses, aspects that remain poorly understood (Irwin et al., 2011; Dalziel et al., 2016; Kormuth et al., 2019; Perlas et al., 2023). However, as noted for reclaimed waters, the presence of viral RNA in these matrices does not necessarily indicate viral infectivity and, consequently, does not directly imply a health risk. This information is crucial for evaluating the risk of transmission through direct or indirect exposure to contaminated aquatic environments, particularly in the context of the current global challenge posed by the widespread circulation of highly pathogenic avian influenza viruses (Charostad et al., 2023).

In contrast, RSV, detected only in a few influent wastewater samples, and SARS-CoV-2 were not detected in any sediment samples, consistent with a previous study in which SARS-CoV-2 was also not detected in sediments (Polo et al., 2021). In line with these observations, somatic coliphages were detected in only one sediment sample at 4.05 ± 0.21 Log_10_ pfu/g, while *E. coli* was not detected in any of the sampling campaigns. These results indicate that *E. coli* and somatic coliphage prevalence was higher in surface waters than in sediments, consistent with Mackowiak et al. (2018) and Martín-Díaz et al. (2020).

Among viral indicators, PMMoV and crAssphage were the most frequently detected in sediment samples, with average concentrations of 2.54 ± 0.89 Log_10_ gc/g (16/17) and 4.58 ± 0.53 Log_10_ gc/g (12/17), respectively.

This baseline information, derived from comprehensive data collected in surface waters and sediments from the Albufera Natural Park, is critical for assessing future changes. It provides a valuable reference point for evaluating the impact of extreme weather events, such as the DANA that occurred in October 2024, which significantly affected the Albufera Natural Park (Mas-Coma et al., 2025).

### Assessment of Viral and Faecal Pollution in the Ebro River Region Ecosystem

A total of 27 samples (15 surface waters and 12 sediments) were collected during three sampling campaign spanning one year to assess the presence of human enteric viruses (HuNoV GI and GII, RV, HAstV, HEV and HAV) and faecal indicators (Fig. 2 and Suppl. Table S15 and S16).

Surface waters collected from the Ebro River region (Suppl. Table S15) exhibited higher prevalence of human enteric viruses (60.00%), compared to Albufera Natural Park (33.33%), indicating elevated viral pollution, with titres ranging from 3.12 to 6.00 Log_10_ gc/L (Fig. 2). HuNoV GII was the most abundant human enteric virus, detected at 5.39 ± 0.19 Log_10_ gc/L (9/15), followed by RV at 5.70 ± 0.36 Log_10_ gc/L in 4 of 15 water samples. HAstV reached titres of 4.63 ± 0.99 Log_10_ gc/L (4/15), while HuNoV GI was detected in two samples at 4.15 ± 0.15 Log_10_ gc/L. HEV was detected only during the colder sampling period, with titres of 3.42 ± 0.36 Log_10_ gc/L (5/15). These findings are consistent with previous studies, where Rusiñol et al. (2015), Jurado et al. (2019) and Forés et al. (2022) reported concentrations of 10^1^–10^3^ gc/L for HuNoV GI and up to 10⁴ gc/L for HuNoV GII in river samples from Llobregat and Besòs river. Regarding respiratory viruses, only SARS-CoV-2 was detected in two samples, with a mean concentration of 3.72 ± 0.11 Log_10_ gc/L.

It is noteworthy that, despite extensive research on chemical pollutants in the Ebro region (Peris et al., 2022; Dulsat-Masvidal et al., 2023; Castaño-Ortiz et al., 2024; Garcia-Torné et al., 2025) and the Albufera Natural Park (Soriano et al., 2023, 2024) their surface waters have yet to be explored from a microbiological perspective, particularly with regard to viral pollution, as has already been done in rivers located on the outskirts of Barcelona (Sano et al., 2010; Pérez-Sautu et al., 2012; Rusiñol et al., 2015; Jurado et al., 2019; Forés et al., 2022; Mejías-Molina et al., 2024; Toribio-Avedillo et al., 2025).

As observed for human enteric virus, Ebro region surface waters exhibited higher prevalence of viral indicators compared to Albufera Natural Park, particularly somatic coliphages at 2.60 ± 0.96 Log_10_ pfu/L (15/15), crAssphage at 4.09 ± 0.49 Log_10_ gc/L (14/15) and PMMoV at 3.33 ± 0.67 Log_10_ gc/L (15/15). Total *E. coli* was detected in 7 of 15 samples, with 2.33 ± 0.90 Log_10_ cfu/L, which were lower levels than those reported by Jurado et al., (2019), ranging from 4.35 to 4.94 Log_10_ cfu/L. Similarly, and consistent with microbiological pollution in Mediterranean rivers, Rusiñol et al. (2015) reported *E. coli* with mean levels of 10^4^ most probable number (MPN)/L in two river sites.

A total of 12 sediment samples (Suppl. Table S16) were collected. The most abundant human enteric virus in the Ebro region, similar to Albufera, was HAstV, with levels of 6.31 ± 1.06 Log_10_ gc/g (3/12), followed by HuNoV GI and GII, with respective values of 5.07 ± 0.22 (5/12) and 6.50 ± 0.79 (2/12) Log_10_ gc/g (Fig. 3). RV showed higher concentrations than the other human enteric viruses but was detected in only one sample (6.65 ± 0.00 Log_10_ gc/g). As in the Albufera, HEV was detected during the winter sampling in one replicate (3.68 Log_10_ gc/g). Although no respiratory viruses were detected in the Ebro River region sediments or waters, IAV was detected in one water and one sediment sample from Albufera Natural Park. This highlights the importance of monitoring respiratory viruses with zoonotic potential, such as influenza viruses, in Mediterranean water bodies, particularly in areas frequented by migratory wild birds, where climatic conditions may facilitate viral transmission and short-term persistence (Ahrens et al., 2022, 2023; Perlas et al., 2023; Filaire et al., 2024). Regarding faecal indicators in sediments, PMMoV was detected in 10 of 12 samples at 2.82 ± 1.07 Log_10_ gc/g and was more frequently recorded than other indicators, although its levels were lower than those of crAssphage, which was detected in 6 of 12 samples at 4.99 ± 1.43 Log_10_ gc/g. Somatic coliphages were detected in 2 of 12 samples at 3.15 ± 1.62 Log_10_ pfu/g, and total *E. coli* was found in 2 of 12 samples at 3.68 ± 0.95 Log_10_ cfu/g.

### Correlation Among Human Enteric Viruses, Faecal Indicators and Physico-Chemical Properties in Surface Water

To further analyse the relationships among human enteric viruses (HuNoV GI and GII, RV, HAstV, HEV and HAV), faecal indicators, and physico-chemical properties in surface waters from both the Albufera Natural Park and Ebro River region, a Spearman correlation analysis was conducted on 66 surface water samples. Analysis of these correlations revealed that PMMoV RT-qPCR signals correlated moderately with HuNoV GII (ρ = 0.42, *p* = 4.8 × 10⁻^4^) and showed a moderate negative relationship with water temperature (ρ = − 0.55, *p* = 1.5 × 10⁻^6^), in line with its temperature-dependent decay reported in both wastewater and freshwater (Kitajima et al., 2018; Dhakar & Geetanjali, 2022; Monleon & Gill, 2024). *E. coli* levels, ranging from 1.18 to 4.10 Log_10_ cfu/L correlated with temperature (ρ = 0.57, *p* = 4.5 × 10⁻^7^) and pH (ρ = 0.44, *p* = 2.4 × 10^4^), while somatic coliphages and crAssphage showed no significant association with any human enteric virus. HAstV levels correlated positively with pH (ρ = 0.44, *p* = 3.5 × 10⁻^4^), consistent with previous observations of virus stability across natural freshwater pH ranges (Gerba, 2007).

Despite no significant correlations were observed with pathogenic human enteric viruses (HuNoV GI and GII, RV, and HAstV), these findings underscore the value of incorporating culturable and faecal indicators (*E. coli* and somatic coliphages) into routine water monitoring to more accurately assess pathogenic risk and guide intervention strategies in both reclaimed and recreational waters, which are also indicators of viability and potential infectivity due to resistance to depuration and disinfection systems. Furthermore, despite the significative positive correlation between PMMoV and HuNoV GII, and considering the influence of agricultural runoff on surface waters, the potential agricultural origin of PMMoV should not be overlooked and warrants further investigation beyond its association with uncontrolled faecal contamination. Nevertheless, pathogen-specific monitoring by (RT)-qPCR or other sensitive techniques remains essential to provide precise and direct information on the presence of harmful organisms.

## Conclusions

The analysed WWTPs achieved substantial *E. coli* reductions (> 5 Log_10_); however, they did not meet the ≥ 6 Log_10_ removal target for somatic coliphages required under Regulation (EC) 2020/741 (European Union, 2020). Consequently, the reclaimed waters were classified as Category B. Moreover, biosolids retained high viral loads, underscoring the urgent need to enhance treatment performance before their reuse.

This study provides the first comprehensive virological assessment of viral contaminant abundance and distribution in two ecologically sensitive Mediterranean ecosystems: the Albufera Natural Park and the Ebro River region. Widespread contamination by human enteric viruses (HuNoV GI and GII, RV, HAstV and HEV) and faecal indicators was observed in both surface waters and sediments, with surface waters from the Ebro River region generally exhibiting higher levels of contamination than those from the Albufera. HuNoV GII, RV, and HEV were frequently detected at high concentrations, with HEV exhibiting marked temporal trends and HuNoV GI and HAstV preferentially accumulating in sediments. Faecal indicators such as PMMoV, crAssphage, and somatic coliphages were consistently detected across all matrices, while *E. coli* and somatic coliphages showed no correlation with viral presence. The detection of zoonotic viruses, including IAV and HEV in Albufera Natural Park, highlights the added value of WES in biodiversity-rich areas.

Although only three sampling campaigns were conducted, one per season, this temporal resolution may not fully capture short-term fluctuations, particularly for low-prevalence or transient viruses. Such limitations could affect detection rates and the viral titers reported. Nevertheless, the consistent patterns observed across both Mediterranean ecosystems and matrices suggest that the findings are broadly representative of the study period. Future investigations with higher-frequency sampling will help refine our understanding of temporal variability and strengthen the robustness of viral surveillance in these ecosystems. A further limitation of this study is that human enteric and respiratory viruses were detected exclusively by RT-qPCR, which does not allow discrimination between infectious and inactivated viral particles. Future work incorporating rapid infectivity proxies, such as cell-culture or capsid integrity–based RT-qPCR approaches, would better improve the assessment of potential public health risk. In addition, the relatively small sediment subsample analysed may not have been fully representative, and the concentrations observed may therefore not reflect those typically found in this type of matrix. Beyond providing valuable data on two previously unstudied geographic areas, this study establishes critical baseline information to inform future interventions. Such baseline data are particularly important in the context of climate-driven extreme weather events, which may exacerbate pathogen mobilisation and dissemination.

## Supplementary Information

Below is the link to the electronic supplementary material.

Supplementary Material 1

Supplementary Material 2

## Data Availability

All data supporting the findings of this study are available within the paper and its Supplementary Information.
