# Supplementary material for: Evaluating Viral Pollution in Wastewater and Mediterranean Ecosystems
Source: Food Environ Virol. 2026 Apr 29;18(2):20. doi: 10.1007/s12560-026-09693-3 (PMC13128697; doi:10.1007/s12560-026-09693-3)
Supplement: Supplementary file 1 — Supplementary Material 1 [file 12560_2026_9693_MOESM1_ESM.pdf]

SUPPLEMENTARY INFORMATION

Evaluating viral pollution in wastewater and Mediterranean ecosystems

Pablo Puchades-Colera[1a], Inés Girón-Guzmán[1a], Enric Cuevas-Ferrando[1], Azahara Díaz-Reolid[1], Irene Falcó[1], Rosa Aznar[2], Marinella Farré[3], Marta Llorca[3], Alba Pérez-Cataluña[1], Gloria Sánchez[1*]

[1]VISAFELab, Department of Preservation and Food Safety Technologies, Institute of Agrochemistry and Food Technology, IATA-CSIC, Av. Agustín Escardino 7, Paterna, Valencia 46980, Spain.

[2]Department of Microbiology and Ecology and Spanish Type Culture Collection (CECT), University of Valencia, MIRRI-ES, Valencia, Spain.

[3]Institute of Environmental Assessment and Water Research, C/Jordi Girona, 18-26, 08034 Barcelona, Spain

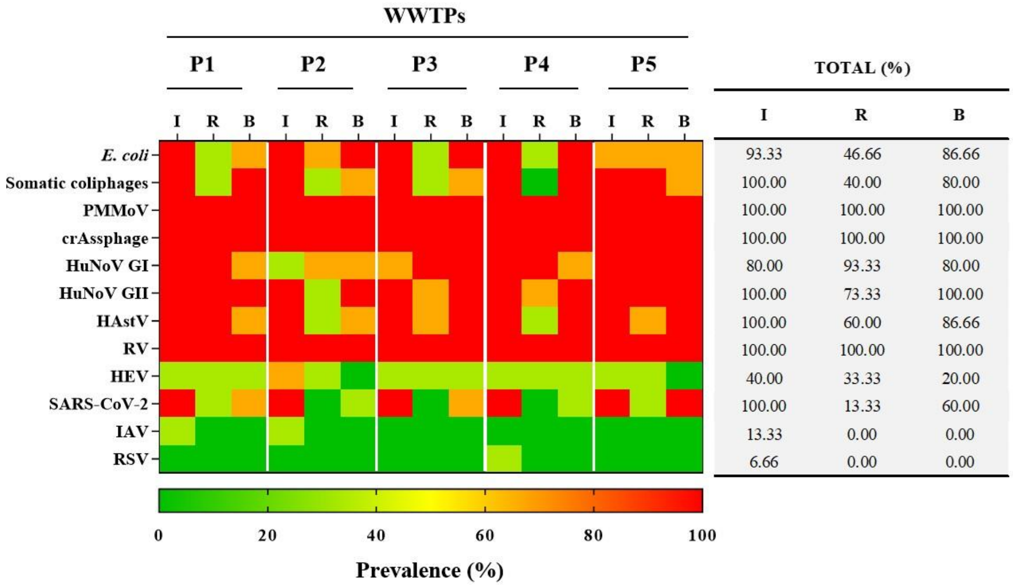

TOTAL (%)

I	R	B
93.33	46.66	86.66
100.00	40.00	80.00
100.00	100.00	100.00
100.00	100.00	100.00
80.00	93.33	80.00
100.00	73.33	100.00
100.00	60.00	86.66
100.00	100.00	100.00
40.00	33.33	20.00
100.00	13.33	60.00
13.33	0.00	0.00
6.66	0.00	0.00

Suppl. Fig. S1 Prevalence of faecal indicators, human enteric viruses and human respiratory viruses in influent wastewater (I), reclaimed water (R) and biosolids (B) samples collected from five different WWTPs (P1-P5) of Albufera Natural Park. Abbreviations: PMMoV, pepper mild mottle virus; HuNoV GI, human norovirus genogroup I; HuNoV GII, human norovirus genogroup II; HAstV, human astrovirus; RV, rotavirus; HEV, hepatitis E virus; SARS-CoV-2, severe acute respiratory syndrome coronavirus 2; IAV, influenza A virus; RSV, respiratory syncytial virus

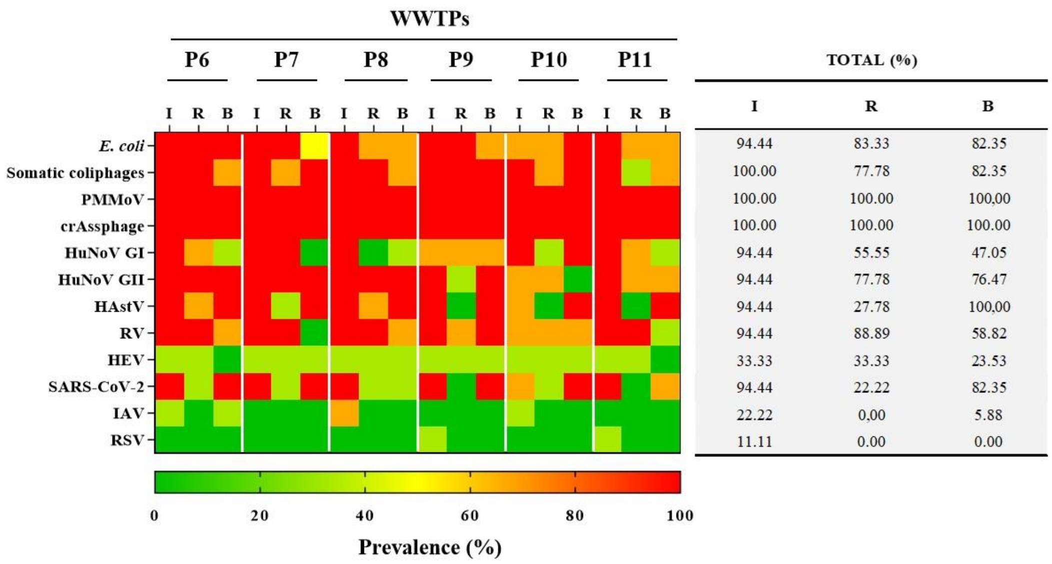

TOTAL (%)		
I	R	B
94.44	83.33	82.35
100.00	77.78	82.35
100.00	100.00	100,00
100.00	100.00	100.00
94.44	55.55	47.05
94.44	77.78	76.47
94.44	27.78	100,00
94.44	88.89	58.82
33.33	33.33	23.53
94.44	22.22	82.35
22.22	0,00	5.88
11.11	0.00	0.00

Suppl. Fig. S2 Prevalence of faecal indicators, human enteric viruses and human respiratory viruses (%) in influent wastewater (I), reclaimed water (R) and biosolids (B) samples collected from five different WWTPs (P6-P11) of Ebro River region. Abbreviations: PMMoV, pepper mild mottle virus; HuNoV GI, human norovirus genogroup I; HuNoV GII, human norovirus genogroup II; HAstV, human astrovirus; RV, rotavirus; HEV, hepatitis E virus; SARS-CoV-2, severe acute respiratory syndrome coronavirus 2; IAV, influenza A virus; RSV, respiratory syncytial virus; gc, genome copies; pfu, plate forming units; cfu, colony forming units

Suppl. Table S1 Information of Albufera Natural Park sampling points: sample type, period, physico-chemical parameters and coordinates. Abbreviations: Nd, Not determined

Sampling points	Sample type	Period	Physico-chemical parameters				Coordinates
			Temperature (°C)	DO (mg/L)	Conductivity (µS/cm)	pH	
**P1**	Influent Wastewater Reclaimed Water Biosolid	-	**Nd**				39.4546, -0.4134
**P2**							39.2708, -0.4029
**P3**							39.2742, -0.2810
**P4**							39.3629, -0.3281
**P5**							39.4423, -0.3442
**SW/SD 1**	Surface Water Sediment	January 22'	13.6	11.64	1493	6.76	39.4221, -0.3530
		July 22'	25.6	17.09	2105	8.33	
		March 23'	16.7	5.86	2230	7.99	
**SW/SD 2**		January 22'	11.3	13.68	2181	6.67	39.3060, -0.3588
		July 22'	28.2	6.69	1439	7.78	
		March 23'	17.9	11.11	1707	8.03	
**SW/SD 3**		January 22'	9.7	11.39	2918	6.83	39.3939, -0.3506
		July 22'	31.4	13.47	2144	7.84	
		March 23'	17.1	13.47	3555	8.00	
**SW/SD 4**		January 22'	9.3	14.79	2398	6.77	39.3814, -0.3397
		July 22'	28.2	6.69	1439	7.87	
		March 23'	22.0	10.03	2145	8.00	
**SW/SD 5**		January 22'	10.6	14.26	1706	6.69	39.3252, -0.3188
		July 22'	28.6	8.45	1806	7.98	
		March 23'	17.1	8.49	1442	8.02	
**SW/SD 6**		January 22'	9.8	14.79	1897	7.20	39.3252, -0.3188
		July 22'	29.2	8.52	1673	7.87	
		March 23'	19.3	11.35	1630	8.03	

Suppl. Table S2 Information of Ebro River region sampling points: sample type, period, physico-chemical parameters and coordinates. Abbreviations: Nd, Not determined

Sampling points	Sample type	Period	Physico-chemical parameters				Coordinates
			Temperature (°C)	DO (mg/L)	Conductivity (µS/cm)	pH	
**P6**	Influent Wastewater Reclaimed Water Biosolid	-	Nd				41.609941, -0.810948
**P7**							41.667902, -0.926062
**P8**							41.239129, 0.551910
**P9**							40.796114, 0.508704
**P10**							40.783837, 0.610137
**P11**							40.627071, 0.622038
**SW/SD 7**	Surface Water Sediment	January 22’	7.6	28.8	1037	6.24	41.581299, -0.760028
		July 22’	27.0	6.66	2420	7.62	
		March 23’	14.6	9.08	1207	8.24	
**SW/SD 8**		January 22’	7.4	14.60	1169	6.68	41.650499, -0.855215
		July 22’	25.1	7.59	2165	8.18	
		March 23’	13.2	10.43	1250	8.00	
**SW/SD 9**		January 22’	7.3	19.71	981	5.84	41.245315, 0.556073
		July 22’	24.7	7.42	1175	7.81	
		March 23’	13.4	15.53	1295	7.97	
**SW/SD 10**		January 22’	9.7	12.85	830	7.26	40.774842, 0.534803
		July 22’	26.6	7.60	1230	7.68	
		March 23’	15.8	15.56	1292	8.03	
**SW/SD 11**		January 22’	9.6	16.92	954	6.50	40.704215, 0.619198
		July 22’	28.3	10.15	3063	7.76	
		March 23’	15.6	15.56	1330	7.97	

Suppl. Table S3 Characteristics and wastewater treatments for each analysed WWTP surrounding Albufera Natural Park

		P1	P2	P3	P4	P5
**Population equivalents**		133.351	94.515	45.783	4.102	664.185
**Flow (m^3/day)**		29.364	21.303	2.200	1.708	200.484
**Pretreatment**	Coarse screening	X	X		X	X
	Fine screening	X	X	X		X
	Flow homogenization tank	X	X			
	Grift removal	X	X	X	X	X
	Grease removal	X	X	X	X	X
**Primary treatment**	Physicochemical treatment	X				X
	Decantation	X	X			X
	Activated sludge	X	X	X	X	X
**Secondary treatment**	Extended aeration	X	X	X	X	X
	Nitrogen removal	X	X	X	X	X
	Phosphor removal	X	X	X	X	X
**Tertiary treatment**	Coagulation-Flocculation	X		X		X
	Filtration	X				X
	UV	X		X	X	X
	Chlorination		X			

Suppl. Table S4 Characteristics and wastewater treatments for each analysed WWTP surrounding Ebro River region

		P6	P7	P8	P9	P10	P11
**Population equivalents**		1.200.000	100.000	12.928	46.847	27.500	28.291
**Flow (m^3/day)**		259.000	34.700	1.943	10.296	5.500	6.310
**Pretreatment**	Coarse screening	X	X	X	X	X	X
	Fine screening	X	X	X	X	X	X
	Flow homogenization tank	X	X		X	X	X
	Grift removal	X	X	X	X	X	X
	Grease removal	X	X		X	X	X
**Primary treatment**	Physicochemical treatment	X	X	X	X	X	X
	Decantation	X	X	X	X	X	X
	Activated sludge	X	X	X	X	X	X
**Secondary treatment**	Extended aeration	X	X	X	X	X	X
	Nitrogen removal				X	X	X
	Phosphor removal	X	X	X	X	X	X
**Tertiary treatment**	Coagulation-Flocculation						
	Filtration						
	UV				X	X	X
	Chlorination	X					

Suppl. Table S5 Primers, probes, PCR conditions, limit of quantification (LoQ/reaction) and limit of detection (LoD/reaction) for all targeted viruses in this work. Abbreviations: PMMoV, pepper mild mottle virus; HuNoV GI, human norovirus genogroup I; HuNoV GII, human norovirus genogroup II; HAV, hepatitis A virus; HEV, hepatitis E virus; HAstV, human astrovirus; RV, rotavirus A; PEDV, porcine epidemic diarrhoea virus; MgV, mengovirus; SARS-CoV-2, severe acute respiratory syndrome coronavirus; RSV, respiratory syncytial virus; IAV, influenza A virus; LoQ/reaction, limit of quantification per reaction; LoD/reaction, limit of detection per reaction

Virus	Primers + probe	Sequence (5' --> 3')	PCR conditions	LoQ/reaction	LoD/reaction	Reference
CrAssphage	064F1	TGTATAGATGCTGCTGCAACTGTACTC	95 ºC, 30 sec; [95 ºC, 05 sec ; 60 ºC, 30 sec] x45 cycles	$1.23x10^2$	37.02	Stachler et al. (2017)
	064R1	CGTTGTTTTCATCTTTATCTTGTCCAT				
	064P	6-FAM-CTGAAATTGTTCATAAGCAA-NFQMGB				
PMMoV		COMERCIAL KIT (PMMoV Fecal Indicator RT-qPCR Kit)	45 ºC, 15 min (RT); 95 ºC, 2 min; [95 ºC, 15 sec; 60 ºC, 60 sec] x45 cyc	1.46	1.06	PMMoV Fecal Indicator RT-qPCR Kit
HuNoV GI	QNIF4	CGCTGGATGCGNTTCCAT	55 ºC, 20 min (RT); 95 ºC, 2 min; [95 ºC, 10 sec; 60 ºC, 30 sec] x45 cyc	6.58	2.08	ISO 15216-1:2017
	NV1LCR	CCTTAGACGCCATCATCATTTAC				
	TM9	6-FAM-TGGACAGGAGATCGC-NFQMGB				
HuNoV GII	QNIF2	ATGTTCAGRTGGATGAGRTTCTCWGA	55 ºC, 20 min (RT); 95 ºC, 2 min; [95 ºC, 10 sec; 60 ºC, 30 sec] x45 cyc	$2.50x10^3$	$1.88x10^3$	ISO 15216-1:2017
	COG2R	TCGACGCCATCTTCATTCACA				
	QNIFs	6-FAM-AGCACGTGGGAGGGCGATCG-3HBQ_1				
HAV	HAV68	TCACCGCCGTTTGCCTAG	55 ºC, 20 min (RT); 95 ºC, 2 min; [95 ºC, 10 sec; 60 ºC, 30 sec] x45 cyc	19.33	9.47	ISO 15216-1:2017
	HAV240	GGAGAGCCCTGGAAGAAA				
	HAV150	6-FAM-CCTGAACCTGCAGGAATTAA-NFQMGB				
HEV	JHRVF	GGTGGTTTCTGGGGTGAC	50 ºC, 30 min (RT); 95 ºC, 15 min; [95 ºC, 10 sec; 55 ºC, 20 sec; 72 ºC,	22.44	9.01	Jothikumar et al. (2006)
	JHEVR	AGGGGTTGGTTGGATGAA				
	JHEVPmod	6-FAM-TGATTCTCAGCCCTTCGC-NFQMGB				
HAstV	AstVorf1b+	AAGCAGCTTCGTGACTCTGG	55 ºC, 20 min (RT); 95 ºC, 2 min; [95 ºC, 10 sec; 60 ºC, 30 sec] x45 cyc	$1.30x10^2$	80.06	Sano et al. (2009)
	AstVorf1b-	AGCCATCACACTTCTTTGGTC				
	AstVorf1BP	6-FAM-AGAGCAACTCCATCGCATTT-3BHQ				
RV	JVKF	CAGTGGTTGATGCTCAAGATGGA	55 ºC, 20 min (RT); 95 ºC, 2 min; [94 ºC, 10 sec; 55 ºC, 30 sec] x45 cyc	$3.01x10^2$	$2.68x10^2$	Jothikumar et al. (2009)
	JVKR	TCATTGTAATCATATTGAATACCCA				
	JVKP	6-FAM-ACAACTGCAGCTTCAAAAGAAGWGT-3BHQ				
PEDV	Forward	CAGGACACATTCTTGGTGGTCTT	42 ºC, 15 min (RT); 95 ºC, 2 min; [95 ºC, 15 sec; 60 ºC, 60 sec] x45 cyc	15.54	10.96	Zhou et al. (2017)
	Reverse	CAAGCAATGTACCACTAAGGAGTGTT				
	Probe	6-FAM-ACGCGCTTCTCACTAC-NFQMGB				
MgV	Mengo110	GCGGGTCCTGCCGAAAGT	42 ºC, 15 min (RT); 95 ºC, 2 min; [95 ºC, 15 sec; 60 ºC, 60 sec] x45 cyc	73.65	51.19	ISO 15216-1:2017
	Mengo209	GAAGTAACATATAGACAGACGCACAC				
	Mengo147	6-FAM-ATCACATTACTGGCCGAAGC-NFQMGB				
SARS-CoV-2 (N1)		COMERCIAL KIT (2019-nCoV_N1 Combined Primer/Probe Mix,	50ºC, 15 min (RT); 95ºC, 2 min; [95ºC, 30 sec; 60ºC, 30 sec] x45 cycles	12.69	8.14	CDC (2020)
RSV	Forward	CACWGAAGATGCWAATCATAAATTCA	50ºC, 20 min (RT); 95ºC, 15 min; [95ºC, 45 sec; 56ºC, 75 sec] x45 cycle	12.87	3.05	Sanghavi et al. (2011)
	Reverse	GTATYTTTATRGTGTCTTCYCTTCCTAACC				
	Probe	6-FAM-TAATAGGTA/ZEN/TGTTATATGCKATGTC-3IABkFQ				
IAV	Forward 1	CAAGACCAATCYTGTCACCTCTGAC	50ºC, 30 min (RT); 95ºC, 2 min; [95ºC, 15 sec; 55ºC, 30 sec] x45 cycles	$1.44x10^3$	$1.27x10^3$	CDC (2021)
	Forward 2	CAAGACCAATYCTGTCACCTYTGAC				
	Reverse 1	CGATTYTGGACAAAVCGTCTACG				
	Reverse 2	GCATTTTGGATAAAGCGTCTACG				
	Probe	6-FAM-TGCAGTCCT/ZEN/CGCTCACTGGGCACG-3IABkFQ				

Suppl. Table S6 Primers, location and PCR conditions of typing nested PCR for HEV

PCR Reaction	Primers	Sequence (5’ → 3’)	Location	Reference
**Reaction 1**	Reverse (HEV-orf2-ro-ch)	GAR AAi GGR CGi GAi GGR GCi GG	6510-6488	Boxman et al. (2017)
	Forward (HEV-orf2-fo-ch)	AAY CAR GGi TGG CGY TCi GTi GAR AC	5885-5910	
**Reaction 2**	Forward (HEV-orf2-fi-ch)	GAG GAG GAA GCT ACC TCY GGY YTi GTi ATG CTY TGY AT	5924-5961	
	Reverse (HEV-orf2-ri-ch)	GGA GAA GGA GTT GGT CGR TCY TGY TCR TGY TGR TT	6489-6455	

Suppl. Table S7 Levels faecal indicators, enteric viruses, respiratory viruses and mean recoveries of PEDV and MgV of influent wastewater samples from wastewater treatment plants in proximity to Albufera Natural Park. Abbreviations: PMMoV, pepper mild mottle virus; HuNoV GI, human norovirus genogroup I; HuNoV GII, human norovirus genogroup II; HAstV, human astrovirus; RV, rotavirus A; HEV, hepatitis E virus; SARS-CoV-2, severe acute respiratory syndrome coronavirus 2; IAV, influenza A virus; RSV, respiratory syncytial virus; PEDV, porcine epidemic diarrhoea virus; MgV, mengovirus; pfu, plaque forming units; cfu, colony forming units; gc, genome copies. *one replicate analysed in this sampling campaign. [a]Expressed in (Log10 pfu/L); [b]Expressed in (Log10 cfu/L); [c]Expressed in (Log10 gc/L)

		Faecal indicators				Enteric viruses (Log_{10} gc/L)					Respiratory viruses (Log_{10} gc/L)			Process control viruses (% recovery)	
		**Somatic coliphages[a]**	**Total *E. coli*[b]**	**PMMoV[c]**	**crAssphage[c]**	**HuNoV GI**	**HuNoV GII**	**HAstV**	**RV**	**HEV**	**SARS-CoV-2**	**IAV**	**RSV**	**PEDV**	**MgV**
**January-22**	**P1**	7.54 ± 0.07	7.46*	6.07 ± 0.00	7.83 ± 0.06	4.21	7.48 ± 0.01	7.07 ± 0.03	7.67 ± 0.00	6.11 ± 0.03	6.12 ± 0.17	<LoD	<LoD	44.52	4.81
	**P2**	6.51 ± 0.04	6.48*	5.95 ± 0.02	7.77 ± 0.02	<LoD	7.31 ± 0.21	6.84 ± 0.04	7.33 ± 0.08	6.05	5.48 ± 0.08	<LoD	<LoD	12.54	16.44
	**P3**	7.04 ± 0.05	7.34*	5.95 ± 0.02	8.28 ± 0.16	<LoD	7.30 ± 0.02	7.06 ± 0.11	7.61 ± 0.01	5.29 ± 0.16	5.95 ± 0.00	<LoD	<LoD	14.74	10.23
	**P4**	6.78 ± 0.03	7.23*	6.15 ± 0.00	8.56 ± 0.07	4.43	7.80 ± 0.02	7.12 ± 0.02	7.27 ± 0.01	6.54 ± 1.43	5.42 ± 0.16	<LoD	4.75	13.44	11.75
	**P5**	7.34 ± 0.23	7.43*	6.30 ± 0.02	8.42 ± 0.04	4.22 ± 0.21	7.82 ± 0.02	7.28 ± 0.01	7.69 ± 0.02	5.41	5.92 ± 0.05	<LoD	<LoD	22.51	11.42
**July-22**	**P1**	7.76 ± 0.20	7.45 ± 0.17	4.94 ± 0.03	7.92 ± 0.06	4.00 ± 0.19	7.27 ± 0.07	7.44 ± 0.02	7.41 ± 0.02	<LoD	5.60 ± 0.12	<LoD	<LoD	32.91	5.11
	**P2**	7.39 ± 0.06	7.13 ± 0.27	4.37 ± 0.01	7.62 ± 0.05	4.04 ± 0.02	7.18 ± 0.02	7.09	7.10	3.52	5.14 ± 0.17	<LoD	<LoD	23.78	31.37
	**P3**	7.33 ± 0.07	8.16 ± 0.04	5.64 ± 0.27	8.56 ± 0.31	4.06 ± 0.13	7.51 ± 0.01	7.50 ± 0.12	7.56 ± 0.03	<LoD	5.58 ± 0.06	<LoD	<LoD	32.45	5.06
	**P4**	7.02 ± 0.02	7.43 ± 0.09	4.89 ± 0.03	7.65 ± 0.05	3.96 ± 0.16	7.24 ± 0.08	7.12 ± 0.04	7.35	<LoD	5.08 ± 0.08	<LoD	<LoD	63.05	83.07
	**P5**	6.77 ± 0.02	7.82 ± 0.19	4.96 ± 0.23	7.31 ± 0.00	4.08 ± 0.44	7.17 ± 0.09	7.14 ± 0.00	7.15 ± 0.00	<LoD	5.19 ± 0.05	<LoD	<LoD	55.35	33.21
**March-23**	**P1**	5.95 ± 0.21	7.53 ± 0.00	5.54 ± 0.07	8.10 ± 0.31	2.81	7.39 ± 0.00	7.67 ± 0.01	7.80 ± 0.00	<LoD	5.16 ± 0.45	6.68	<LoD	6.91	13.51
	**P2**	5.30 ± 0.00	6.70 ± 0.00	5.30 ± 0.01	7.78 ± 0.17	<LoD	7.02 ± 0.06	7.79 ± 0.04	7.58 ± 0.05	<LoD	5.02 ± 0.07	6.42	<LoD	21.79	15.06
	**P3**	4.77 ± 0.05	7.33 ± 0.04	5.46 ± 0.01	7.64 ± 0.06	4.10 ± 0.16	7.00 ± 0.02	7.68 ± 0.03	7.71 ± 0.05	<LoD	4.72 ± 0.03	<LoD	<LoD	20.47	18.33
	**P4**	5.48 ± 0.00	7.65 ± 0.07	5.73 ± 0.10	7.74 ± 0.07	3.42 ± 0.13	7.17 ± 0.08	7.68 ± 0.09	7.91 ± 0.02	<LoD	4.74 ± 0.00	<LoD	<LoD	14.96	10.28
	**P5**	5.40 ± 0.12	6.60 ± 0.16	5.55 ± 0.10	7.82 ± 0.10	4.03 ± 0.14	8.06 ± 0.03	8.06 ± 0.02	7.93 ± 0.01	<LoD	5.28 ± 0.02	<LoD	<LoD	29.33	9.62

Suppl. Table S8 Levels of faecal indicators, enteric viruses, respiratory viruses and mean recoveries of PEDV and MgV of influent wastewater samples from wastewater treatment plants in proximity to Ebro River region. Abbreviations: PMMoV, pepper mild mottle virus; HuNoV GI, human norovirus genogroup I; HuNoV GII, human norovirus genogroup II; HAstV, human astrovirus; RV, rotavirus A; HEV, hepatitis E virus; SARS-CoV-2, severe acute respiratory syndrome coronavirus 2; IAV, influenza A virus; RSV, respiratory syncytial virus; PEDV, porcine epidemic diarrhoea virus; MgV, mengovirus; pfu, plaque forming units; cfu, colony forming units; gc, genome copies. *one replicate analysed in this sampling campaign. [a]Expressed in (Log10 pfu/L); [b]Expressed in (Log10 cfu/L); [c]Expressed in (Log10 gc/L)

		Faecal indicators				Enteric viruses (Log_{10} gc/L)					Respiratory viruses (Log_{10} gc/L)			Process control viruses (% recovery)	
		Somatic coliphages[a]	Total *E. coli*[b]	PMMoV[c]	crAssphage[c]	HuNoV GI	HuNoV GII	HAstV	RV	HEV	SARS-CoV-2	IAV	RSV	PEDV	MgV
January-22	P6	7.36 ± 0.08	8.29*	6.43 ± 0.01	8.85 ± 0.11	3.67 ± 0.34	7.88 ± 0.01	7.53 ± 0.13	6.91 ± 0.05	5.14 ± 0.11	6.31 ± 0.00	<LoD	<LoD	26.34	5.80
	P7	6.97 ± 0.03	7.32*	6.22 ± 0.10	8.58 ± 0.06	2.80	7.58 ± 0.04	7.32 ± 0.08	7.36 ± 0.16	5.00	6.19 ± 0.09	<LoD	<LoD	47.55	19.74
	P8	6.93 ± 0.03	7.18*	6.52 ± 0.05	8.67 ± 0.17	3.68 ± 0.48	7.68 ± 0.01	7.82 ± 0.07	7.70 ± 0.03	4.75	6.22 ± 0.04	6.44	<LoD	12.52	4.59
	P9	7.54 ± 0.05	7.52*	6.22 ± 0.00	8.22 ± 0.03	<LoD	7.34 ± 0.12	7.46 ± 0.01	7.92 ± 0.04	4.89 ± 0.53	6.08 ± 0.03	<LoD	4.48	45.50	8.77
	P10	7.45 ± 0.11	7.38*	5.80 ± 0.02	8.25 ± 0.02	3.54 ± 0.13	7.37 ± 0.08	6.84 ± 0.01	7.64 ± 0.20	5.04	6.12 ± 0.31	<LoD	<LoD	33.79	4.80
	P11	6.65 ± 0.03	7.38*	5.81 ± 0.03	8.01 ± 0.14	3.01	8.09 ± 0.17	7.33 ± 0.02	7.84 ± 0.40	5.03 ± 0.11	6.04 ± 0.12	<LoD	5.08 ± 0.20	32.73	11.25
July-22	P6	7.54 ± 0.12	7.77 ± 0.04	5.22 ± 0.13	8.43 ± 0.01	3.68 ± 0.38	7.19 ± 0.08	7.63 ± 0.00	7.04 ± 0.14	<LoD	5.57 ± 0.07	<LoD	<LoD	16.84	12.52
	P7	7.47 ± 0.03	7.65 ± 0.09	5.59 ± 0.70	7.98 ± 0.13	3.93 ± 0.04	7.13 ± 0.12	7.37 ± 0.05	8.29 ± 0.01	<LoD	5.27 ± 0.15	<LoD	<LoD	57.58	72.86
	P8	7.30 ± 0.09	6.40 ± 0.43	5.43 ± 0.14	7.54 ± 0.03	3.62	6.97 ± 0.02	7.53 ± 0.07	7.03 ± 0.03	<LoD	5.07 ± 0.09	<LoD	<LoD	77.20	43.62
	P9	4.19 ± 0.06	7.53 ± 0.02	4.98 ± 0.09	8.32 ± 0.01	3.82	7.06 ± 0.06	7.46 ± 0.01	7.40 ± 0.07	<LoD	5.59 ± 0.11	<LoD	<LoD	60.90	6.14
	P10	7.48 ± 0.05	7.00	2.83 ± 0.06	8.57 ± 0.04	3.46	<LoD	<LoD	<LoD	<LoD	<LoD	<LoD	<LoD	78.00	20.95
	P11	8.16 ± 0.07	6.70	5.30 ± 0.17	8.62 ± 0.04	4.15 ± 0.01	7.01 ± 0.06	7.61 ± 0.05	7.02 ± 0.00	<LoD	5.48 ± 0.02	<LoD	<LoD	56.22	22.26
March-23	P6	5.18 ± 0.21	6.74 ± 0.17	4.77 ± 0.03	7.90 ± 0.14	3.70 ± 0.00	7.43 ± 0.05	7.97 ± 0.00	7.68 ± 0.01	<LoD	5.20 ± 0.09	6.14	<LoD	15.19	4.26
	P7	5.95 ± 0.14	6.48 ± 0.00	6.10 ± 0.25	8.25 ± 0.06	3.90 ± 0.05	7.48 ± 0.40	8.18 ± 0.01	8.17 ± 0.06	<LoD	5.44 ± 0.44	<LoD	<LoD	14.16	8.31
	P8	6.74 ± 0.17	6.00 ± 0.00	5.18 ± 0.02	8.01 ± 0.51	3.17	7.11 ± 0.10	7.74 ± 0.01	8.57 ± 0.09	<LoD	5.71 ± 0.11	6.53	<LoD	49.07	13.54
	P9	6.02 ± 0.03	6.18 ± 0.21	5.60 ± 0.06	8.05 ± 0.36	3.34	7.64 ± 0.05	7.94 ± 0.05	8.26 ± 0.03	<LoD	5.29 ± 0.01	<LoD	<LoD	28.92	4.63
	P10	6.18 ± 0.08	6.40 ± 0.12	5.62 ± 0.30	8.76 ± 0.10	3.40	7.62 ± 0.41	7.68 ± 0.01	7.84 ± 0.09	<LoD	5.06 ± 0.38	6.27	<LoD	7.53	3.69
	P11	5.54 ± 0.00	7.55 ± 0.08	5.24 ± 0.06	8.01 ± 0.08	3.40 ± 0.61	7.82 ± 0.33	7.73 ± 0.01	8.55 ± 0.01	<LoD	5.72 ± 0.01	<LoD	<LoD	75.31	5.24

Suppl. Table S9 Levels of faecal indicators, enteric viruses, respiratory viruses and mean recoveries of PEDV and MgV of reclaimed water samples from wastewater treatment plants in proximity to Albufera Natural Park. Abbreviations: PMMoV, pepper mild mottle virus; HuNoV GI, human norovirus genogroup I; HuNoV GII, human norovirus genogroup II; HAstV, human astrovirus; RV, rotavirus A; HEV, hepatitis E virus; SARS-CoV-2, severe acute respiratory syndrome coronavirus 2; IAV, influenza A virus; RSV, respiratory syncytial virus; PEDV, porcine epidemic diarrhoea virus; MgV, mengovirus; pfu, plaque forming units; cfu, colony forming units; gc, genome copies. *one replicate analysed in this sampling campaign. [a]Expressed in (Log10 pfu/L); [b]Expressed in (Log10 cfu/L); [c]Expressed in (Log10 gc/L)

		Faecal indicators				Enteric viruses (Log_{10} gc/L)					Respiratory viruses (Log_{10} gc/L)			Process control viruses (% recovery)	
		**Somatic coliphages[a]**	**Total *E. coli*[b]**	**PMMoV[c]**	**crAssphage[c]**	**HuNoV GI**	**HuNoV GII**	**HAstV**	**RV**	**HEV**	**SARS-CoV-2**	**IAV**	**RSV**	**PEDV**	**MgV**
**January-22**	**P1**	5.89 ± 0.04	<LoD	5.38 ± 0.06	6.09 ± 0.11	2.88	6.56 ± 0.09	5.75 ± 0.02	7.40 ± 0.01	5.22	<LoD	<LoD	<LoD	8.35	24.17
	**P2**	<LoD	1.18*	3.64 ± 0.01	5.07 ± 0.10	3.03 ± 0.08	6.09	<LoD	7.34 ± 0.05	5.04 ± 0.37	<LoD	<LoD	<LoD	26.93	42.65
	**P3**	<LoD	<LoD	4.42 ± 0.02	5.59 ± 0.01	3.24	<LoD	<LoD	6.59 ± 0.18	4.93	<LoD	<LoD	<LoD	4.74	16.51
	**P4**	<LoD	<LoD	4.27 ± 0.12	4.69 ± 0.11	2.88 ± 0.02	6.06	<LoD	6.78 ± 0.15	4.22	<LoD	<LoD	<LoD	15.12	63.43
	**P5**	5.76 ± 0.10	2.54*	5.19 ± 0.06	6.92 ± 0.06	2.83 ± 0.03	7.07 ± 0.03	6.10 ± 0.10	8.06 ± 0.07	4.56	4.60 ± 0.25	<LoD	<LoD	8.68	5.08
**July-22**	**P1**	<LoD	<LoD	4.52 ± 0.03	5.79 ± 0.05	3.24	6.40 ± 0.15	6.93	7.14 ± 0.04	<LoD	4.36	<LoD	<LoD	46.23	43.99
	**P2**	4.24 ± 0.02	0.88	2.53 ± 0.07	<LoD	<LoD	<LoD	<LoD	6.53	<LoD	<LoD	<LoD	<LoD	5.27	19.43
	**P3**	<LoD	<LoD	3.67 ± 0.32	4.87 ± 0.10	3.32	6.05	5.27	7.03	<LoD	<LoD	<LoD	<LoD	17.36	8.86
	**P4**	<LoD	2.87 ± 0.31	3.75 ± 0.06	4.36	2.91	5.99	<LoD	7.44 ± 0.15	<LoD	<LoD	<LoD	<LoD	36.13	11.99
	**P5**	5.50 ± 0.03	<LoD	3.95 ± 0.41	6.05 ± 0.10	3.28 ± 0.07	5.99	<LoD	7.09 ± 0.06	<LoD	<LoD	<LoD	<LoD	13.49	19.96
**March-23**	**P1**	<LoD	0.70*	4.51 ± 0.04	5.80 ± 0.17	2.78	6.10 ± 0.16	6.55 ± 0.01	7.91 ± 0.02	<LoD	<LoD	<LoD	<LoD	25.82	54.84
	**P2**	<LoD	<LoD	3.59 ± 0.03	5.30 ± 0.09	3.53	<LoD	6.77 ± 0.00	7.21 ± 0.01	<LoD	<LoD	<LoD	<LoD	14.47	30.67
	**P3**	<LoD	1.00*	4.29 ± 0.15	5.61 ± 0.16	3.06	6.29	6.54 ± 0.07	7.05 ± 0.03	<LoD	<LoD	<LoD	<LoD	22.83	59.61
	**P4**	<LoD	<LoD	4.29 ± 0.03	4.36 ± 0.35	3.69	<LoD	6.42 ± 0.02	7.83 ± 0.00	<LoD	<LoD	<LoD	<LoD	16.11	58.53
	**P5**	4.18 ± 0.00	3.18*	5.24 ± 0.03	6.85 ± 0.09	3.42 ± 0.29	6.66 ± 0.08	6.75 ± 0.01	8.57 ± 0.04	<LoD	<LoD	<LoD	<LoD	22.20	64.71

Suppl. Table S10 Levels of faecal indicators, enteric viruses, respiratory viruses and mean recoveries of PEDV and MgV of reclaimed water samples from wastewater treatment plants in proximity to Ebro River region. Abbreviations: PMMoV, pepper mild mottle virus; HuNoV GI, human norovirus genogroup I; HuNoV GII, human norovirus genogroup II; HAstV, human astrovirus; RV, rotavirus A; HEV, hepatitis E virus; SARS-CoV-2, severe acute respiratory syndrome coronavirus 2; IAV, influenza A virus; RSV, respiratory syncytial virus; PEDV, porcine epidemic diarrhoea virus; MgV, mengovirus; pfu, plaque forming units; cfu, colony forming units; gc, genome copies. *one replicate analysed in this sampling campaign. [a]Expressed in (Log10 pfu/L); [b]Expressed in (Log10 cfu/L); [c]Expressed in (Log10 gc/L)

		**Faecal indicators**				**Enteric viruses (Log_{10} gc/L)**					**Respiratory viruses (Log_{10} gc/L)**			**Process control viruses (% recovery)**	
		**Somatic coliphages[a]**	**Total *E. coli*[b]**	**PMMoV[c]**	**crAssphage[c]**	**HuNoV GI**	**HuNoV GII**	**HAstV**	**RV**	**HEV**	**SARS-CoV-2**	**IAV**	**RSV**	**PEDV**	**MgV**
**January-22**	**P6**	6.34 ± 0.18	2.26*	5.35 ± 0.05	6.16 ± 0.04	<LoD	6.78 ± 0.05	5.98 ± 0.02	7.24 ± 0.00	6.03 ± 0.43	4.56 ± 0.15	<LoD	<LoD	32.93	48.38
	**P7**	6.46 ± 0.13	1.48 *	5.77 ± 0.06	6.83 ± 0.11	2.87 ± 0.02	7.35 ± 0.04	<LoD	8.28 ± 0.02	4.86 ± 0.51	4.87 ±0.08	<LoD	<LoD	28.02	48.73
	**P8**	5.04 ± 0.10	<LoD	4.90 ± 0.09	5.94 ± 0.11	<LoD	6.25 ± 0.21	<LoD	7.80 ± 0.03	4.30	<LoD	<LoD	<LoD	22.86	53.05
	**P9**	4.83 ± 0.05	1.18*	4.33 ± 0.06	5.15 ± 0.08	<LoD	<LoD	<LoD	6.90 ± 0.05	4.71	<LoD	<LoD	<LoD	57.61	43.39
	**P10**	5.13 ± 0.03	<LoD	5.36 ± 0.07	5.67 ± 0.05	<LoD	6.04 ± 0.07	<LoD	6.47 ± 0.09	5.03 ± 0.87	4.23	<LoD	<LoD	84.78	78.71
	**P11**	<LoD	<LoD	4.94 ± 0.01	5.11 ± 0.15	<LoD	6.45 ± 0.06	<LoD	6.46 ± 0.06	4.20	<LoD	<LoD	<LoD	61.25	65.85
**July-22**	**P6**	3.55 ± 0.04	3.52 ± 0.07	4.58 ± 0.03	6.06 ± 0.05	3.78 ± 0.01	6.56 ± 0.06	7.04 ± 0.25	7.39 ± 0.14	<LoD	<LoD	<LoD	<LoD	67.62	16.14
	**P7**	3.52 ± 0.06	1.24 ± 0.55	4.32 ± 0.05	5.96 ± 0.02	4.05 ± 0.04	6.28 ± 0.27	6.53 ± 0.72	7.19 ± 0.01	<LoD	<LoD	<LoD	<LoD	51.15	24.06
	**P8**	3.47 ± 0.03	2.03 ± 0.01	4.95 ± 0.02	6.32 ± 0.01	<LoD	6.35 ± 0.27	7.13 ± 0.04	7.16 ± 0.03	<LoD	<LoD	<LoD	<LoD	38.87	30.71
	**P9**	3.26 ± 0.05	2.55 ± 0.06	3.61 ± 0.16	5.42 ± 0.03	3.02	5.94 ± 0.07	<LoD	<LoD	<LoD	<LoD	<LoD	<LoD	27.56	67.33
	**P10**	3.19 ± 0.12	0.40	3.20 ± 0.01	4.41	3.28	<LoD	<LoD	<LoD	<LoD	<LoD	<LoD	<LoD	29.37	24.88
	**P11**	3.15 ± 0.02	1.00 ± 0.34	3.53 ± 0.04	5.50 ± 0.10	3.39 ± 0.34	<LoD	<LoD	6.67 ± 0.15	<LoD	<LoD	<LoD	<LoD	76.76	48.08
**March-23**	**P6**	4.74 ± 0.06	3.48*	4.59 ± 0.12	7.25 ± 0.75	3.41 ± 0.03	6.46 ± 0.19	<LoD	7.72 ± 0.02	<LoD	<LoD	<LoD	<LoD	12.36	17.86
	**P7**	<LoD	2.18*	4.40 ± 0.04	5.82 ± 0.07	3.37 ± 0.11	6.23	<LoD	7.31 ± 0.11	<LoD	<LoD	<LoD	<LoD	64.56	80.98
	**P8**	5.32 ± 0.09	3.88*	4.89 ± 0.12	7.08 ± 0.19	<LoD	7.33 ± 0.04	7.69	7.62 ± 0.92	<LoD	4.44	<LoD	<LoD	54.14	69.84
	**P9**	3.00	2.00*	3.79 ± 0.05	5.60 ± 0.47	2.99	<LoD	<LoD	7.59 ± 0.45	<LoD	<LoD	<LoD	<LoD	24.95	74.67
	**P10**	<LoD	1.93*	4.35 ± 0.34	5.28 ± 0.20	<LoD	6.14 ± 0.01	<LoD	7.05 ± 0.20	<LoD	<LoD	<LoD	<LoD	36.04	54.70
	**P11**	<LoD	1.00*	4.91 ± 0.36	5.42 ± 0.02	3.18 ± 0.20	6.52 ± 0.32	<LoD	8.14 ± 0.05	<LoD	<LoD	<LoD	<LoD	50.09	76.90

Suppl. Table S11 Levels of faecal indicators, enteric viruses, respiratory viruses of biosolid samples from wastewater treatment plants in proximity to Albufera Natural Park. Abbreviations: PMMoV, pepper mild mottle virus; HuNoV GI, human norovirus genogroup I; HuNoV GII, human norovirus genogroup II; HAstV, human astrovirus; RV, rotavirus A; HEV, hepatitis E virus; SARS-CoV-2, severe acute respiratory syndrome coronavirus 2; IAV, influenza A virus; RSV, respiratory syncytial virus; pfu, plaque forming units; cfu, colony forming units; gc, genome copies. *one replicate analysed in this sampling campaign. [a]Expressed in (Log10 pfu/g); [b]Expressed in (Log10 cfu/g); [c]Expressed in (Log10 gc/g)

		Faecal indicators				Enteric viruses (Log_{10} gc/g)					Respiratory viruses (Log_{10} gc/g)		
		**Somatic coliphages[a]**	**Total *E. coli*[b]**	**PMMoV[c]**	**crAssphage[c]**	**HuNoV GI**	**HuNoV GII**	**HAstV**	**RV**	**HEV**	**SARS-CoV-2**	**IAV**	**RSV**
**January-22**	**P1**	2.86 ± 0.00	<LoD	5.85 ± 0.20	7.52 ± 0.01	<LoD	6.80 ± 0.33	6.15 ± 0.24	7.14 ± 0.04	4.84 ± 0.00	4.22	<LoD	<LoD
	**P2**	<LoD	3.60*	5.64 ± 0.02	7.15 ± 0.12	6.09	6.78 ± 0.28	6.42 ± 0.02	7.41 ± 0.01	<LoD	4.53 ± 0.07	<LoD	<LoD
	**P3**	1.15 ± 0.21	4.11*	6.14 ± 0.01	7.12 ± 0.02	5.74	7.10 ± 0.04	6.77 ± 0.00	7.17 ± 0.01	4.60 ± 0.00	4.13	<LoD	<LoD
	**P4**	1.63 ± 0.21	3.60*	5.85 ± 0.01	7.49 ± 0.13	<LoD	7.08 ± 0.03	6.92 ± 0.02	7.95 ± 0.01	4.23 ± 0.00	<LoD	<LoD	<LoD
	**P5**	2.59 ± 0.02	<LoD	5.71 ± 0.01	7.52 ± 0.11	5.93	7.07 ± 0.00	6.69 ± 0.06	7.84 ± 0.05	<LoD	4.86 ± 0.01	<LoD	<LoD
**July-22**	**P1**	3.22 ± 0.02	6.13 ± 0.09	4.64 ± 0.28	6.45 ± 0.07	5.45	6.29 ± 0.15	<LoD	6.81 ± 0.15	<LoD	<LoD	<LoD	<LoD
	**P2**	3.93 ± 0.15	4.94 ± 0.34	3.68 ± 0.09	6.31 ± 0.05	<LoD	6.28	<LoD	7.41 ± 0.09	<LoD	<LoD	<LoD	<LoD
	**P3**	5.41 ± 0.05	4.98 ± 0.07	4.88 ± 0.32	6.21 ± 0.03	5.21 ± 0.16	6.46 ± 0.14	7.10	8.05 ± 0.12	<LoD	<LoD	<LoD	<LoD
	**P4**	5.48 ± 0.07	4.97 ± 0.05	4.72 ± 0.21	6.21 ± 0.08	5.08	6.71 ± 0.03	6.99 ± 0.11	7.79 ± 0.00	<LoD	<LoD	<LoD	<LoD
	**P5**	5.31 ± 0.05	4.45 ± 0.38	4.61 ± 0.03	6.55 ± 0.00	5.40	6.68 ± 0.14	6.76 ± 0.19	7.28 ± 0.01	<LoD	4.36 ± 0.14	<LoD	<LoD
**March-23**	**P1**	2.37 ± 0.21	3.69 ± 0.12	4.78 ± 0.03	6.75 ± 0.00	5.19	6.17 ± 0.51	6.71 ± 0.02	7.15 ± 0.18	<LoD	4.15	<LoD	<LoD
	**P2**	2.10 ± 0.00	3.95 ± 0.07	4.10 ± 0.08	6.62 ± 0.36	4.85 ± 0.20	5.89	7.34 ± 0.17	7.77 ± 0.15	<LoD	<LoD	<LoD	<LoD
	**P3**	<LoD	4.74 ± 0.11	5.09 ± 0.05	6.66 ± 0.16	5.21 ± 0.14	6.75 ± 0.36	7.51 ± 0.08	8.05 ± 0.06	<LoD	4.52	<LoD	<LoD
	**P4**	2.30 ± 0.00	4.98 ± 0.02	5.36 ± 0.02	6.51 ± 0.05	5.13 ± 0.48	6.80 ± 0.17	7.65 ± 0.11	7.92 ± 0.04	<LoD	4.32 ± 0.10	<LoD	<LoD
	**P5**	<LoD	4.10 ± 0.14	5.38 ± 0.06	6.93 ± 0.00	5.30	6.64 ± 0.11	6.55 ± 0.01	7.83 ± 0.00	<LoD	4.73	<LoD	<LoD

Suppl. Table S12 Levels of faecal indicators, enteric viruses, respiratory viruses of biosolid samples from wastewater treatment plants in proximity to Ebro River region. Abbreviations: PMMoV, pepper mild mottle virus; HuNoV GI, human norovirus genogroup I; HuNoV GII, human norovirus genogroup II; HAstV, human astrovirus; RV, rotavirus A; HEV, hepatitis E virus; SARS-CoV-2, severe acute respiratory syndrome coronavirus 2; IAV, influenza A virus; RSV, respiratory syncytial virus; pfu, plaque forming units; cfu, colony forming units; gc, genome copies. *one replicate analysed in this sampling campaign. [a]Expressed in (Log10 pfu/g); [b]Expressed in (Log10 cfu/g); [c]Expressed in (Log10 gc/g). Na, not analysed

		**Faecal indicators**				**Enteric viruses (Log_{10} gc/g)**					**Respiratory viruses (Log_{10} gc/g)**		
		**Somatic coliphages[a]**	**Total *E. coli*[b]**	**PMMoV[c]**	**crAssphage[c]**	**HuNoV GI**	**HuNoV GII**	**HAstV**	**RV**	**HEV**	**SARS-CoV-2**	**IAV**	**RSV**
**January-22**	**P6**	3.32 ± 0.02	3.90*	4.59 ± 0.03	7.00 ± 0.11	<LoD	6.71 ± 0.09	6.64 ± 0.01	5.33	<LoD	4.96 ± 0.05	<LoD	<LoD
	**P7**	2.84 ± 0.08	<LoD	5.12 ± 0.03	6.83 ± 0.20	<LoD	6.64 ± 0.08	6.37 ± 0.04	<LoD	4.11	5.37 ± 0.03	<LoD	<LoD
	**P8**	1.74 ± 0.06	<LoD	5.11 ± 0.00	6.49 ± 0.01	<LoD	6.47 ± 0.05	5.98 ± 0.01	<LoD	3.59	<LoD	<LoD	<LoD
	**P9**	3.58 ± 0.03	<LoD	4.73 ± 0.03	6.88 ± 0.01	5.89 ± 0.20	7.03 ± 0.08	7.26 ± 0.01	6.24 ± 0.11	3.47	4.86 ± 0.03	<LoD	<LoD
	**P10**	2.16 ± 0.02	4.56*	5.73 ± 0.01	7.17 ± 0.04	6.36 ± 0.18	7.32 ± 0.00	7.34 ± 0.02	7.83 ± 0.04	4.71	4.77 ± 0.14	<LoD	<LoD
	**P11**	2.78 ± 0.74	<LoD	5.81 ± 0.04	6.75 ± 0.22	5.93	6.91 ± 0.02	6.33 ± 0.03	<LoD	<LoD	4.66	<LoD	<LoD
**July-22**	**P6**	5.34 ± 0.03	5.45 ± 0.10	4.46 ± 0.12	6.92 ± 0.01	<LoD	6.38 ± 0.15	7.41 ± 0.00	<LoD	<LoD	4.74 ± 0.07	6.38	<LoD
	**P7**	3.77 ± 0.02	4.87 ± 0.11	5.23 ± 0.23	6.16 ± 0.03	<LoD	5.88	7.04 ± 0.06	<LoD	<LoD	4.04	<LoD	<LoD
	**P8**	5.83 ± 0.03	4.61 ± 0.06	5.22 ± 0.18	7.13 ± 0.04	<LoD	6.27 ± 0.08	7.21 ± 0.01	7.24	<LoD	4.96 ± 0.06	<LoD	<LoD
	**P9**	5.77 ± 0.01	4.93 ± 0.06	4.59 ± 0.12	6.57 ± 0.05	<LoD	6.08	7.44 ± 0.00	7.32 ± 0.05	<LoD	4.40 ± 0.15	<LoD	<LoD
	**P10**	5.45 ± 0.02	5.62 ± 0.48	5.00 ± 0.07	7.20 ± 0.02	4.73	6.37 ± 0.45	6.77 ± 0.00	<LoD	<LoD	4.11	<LoD	<LoD
	**P11**	5.80 ± 0.01	4.70 ± 0.01	4.67 ± 0.25	6.52 ± 0.02	<LoD	<LoD	7.51 ± 0.00	<LoD	<LoD	4.12	<LoD	<LoD
**March-23**	**P6**	<LoD	5.48 ± 0.00	5.48 ± 0.25	7.47 ± 0.10	6.04 ± 0.25	6.98 ± 0.16	7.84 ± 0.10	7.54 ± 0.46	<LoD	4.34 ± 0.19	<LoD	<LoD
	**P7**	Na				Na					Na		
	**P8**	<LoD	4.04 ± 0.19	4.87 ± 0.07	6.86 ± 0.03	5.32 ± 0.09	7.39 ± 0.29	8.01 ± 0.31	7.36 ± 0.05	<LoD	<LoD	<LoD	<LoD
	**P9**	3.31 ± 0.05	4.50 ± 0.02	4.36 ± 0.02	7.31 ± 0.07	5.91 ± 0.44	6.92 ± 0.07	7.65 ± 0.14	7.99 ± 1.05	<LoD	4.14 ± 0.05	<LoD	<LoD
	**P10**	3.15 ± 0.21	5.27 ± 0.07	5.32 ± 0.44	7.27 ± 0.04	5.31 ± 0.36	7.12 ± 0.02	7.58 ± 0.04	8.32 ± 0.04	<LoD	4.29 ± 0.16	<LoD	<LoD
	**P11**	<LoD	4.60 ± 0.01	5.14 ± 0.03	7.40 ± 0.04	<LoD	7.01	8.11 ± 0.03	8.21 ± 0.05	<LoD	<LoD	<LoD	<LoD

Suppl. Table S13 Levels of faecal indicators, enteric viruses, respiratory viruses and mean recoveries of PEDV and MgV in surface waters of Albufera Natural Park. Abbreviations: PMMoV, pepper mild mottle virus; HuNoV GI, human norovirus genogroup I; HuNoV GII, human norovirus genogroup II; HAstV, human astrovirus; RV, rotavirus A; HEV, hepatitis E virus; SARS-CoV-2, severe acute respiratory syndrome coronavirus 2; IAV, influenza A virus; RSV, respiratory syncytial virus; PEDV, porcine epidemic diarrhoea virus; MgV, mengovirus; pfu, plaque forming units; cfu, colony forming units; gc, genome copies. *one replicate analysed in this sampling campaign. [a]Expressed in (Log10 pfu/L); [b]Expressed in (Log10 cfu/L); [c]Expressed in (Log10 gc/L)

		**Faecal indicators**				**Enteric viruses (Log_{10} gc/L)**					**Respiratory viruses (Log_{10} gc/L)**			**Process control viruses (% recovery)**	
		**Somatic coliphages**[a]	**Total *E. coli***[b]	**PMMoV**[c]	**crAssphage**[c]	**HuNoV GI**	**HuNoV GII**	**HAstV**	**RV**	**HEV**	**SARS-CoV-2**	**IAV**	**RSV**	**PEDV**	**MgV**
**January-22**	**SW1**	1.30 ± 0.06	<LoD	4.81 ± 0.04	5.98 ± 0.00	<LoD	<LoD	<LoD	6.84 ± 0.11	<LoD	3.22	<LoD	<LoD	25.56	3.84
	**SW2**	0.71 ± 0.09	<LoD	2.76 ± 0.06	<LoD	<LoD	5.04	<LoD	<LoD	4.55 ± 0.12	<LoD	<LoD	<LoD	17.88	3.15
	**SW3**	2.52 ± 0.02	<LoD	4.50 ± 0.08	4.07 ± 0.03	<LoD	5.07 ± 0.10	<LoD	<LoD	3.50 ± 0.41	<LoD	<LoD	<LoD	18.80	1.89
	**SW4**	1.45 ± 0.02	<LoD	4.15 ± 0.11	3.70 ± 0.02	<LoD	<LoD	<LoD	<LoD	2.52	<LoD	<LoD	<LoD	8.47	5.49
	**SW5**	1.58 ± 0.16	<LoD	3.30 ± 0.15	3.54 ± 0.08	<LoD	<LoD	<LoD	<LoD	<LoD	<LoD	<LoD	<LoD	8.52	5.18
	**SW6**	0.86 ± 0.21	2*	2.01 ± 0.00	<LoD	<LoD	<LoD	<LoD	<LoD	3.22 ± 0.12	<LoD	<LoD	<LoD	8.04	6.21
**July-22**	**SW1**	3.28 ± 0.12	3.66 ±0.07	3.74 ± 0.01	4.62 ± 0.10	<LoD	<LoD	<LoD	<LoD	<LoD	<LoD	<LoD	<LoD	3.47	25.53
	**SW2**	2.57 ± 0.08	2.46 ± 0.03	2.14 ± 0.14	3.31 ± 0.22	<LoD	<LoD	<LoD	<LoD	<LoD	<LoD	<LoD	<LoD	2.79	4.68
	**SW3**	2.17 ± 0.01	1.95 ± 0.10	2.68 ± 0.05	<LoD	<LoD	4.94	<LoD	<LoD	<LoD	<LoD	<LoD	<LoD	2.14	20.53
	**SW4**	1.55 ± 0.05	2.10 ± 0.43	3.18 ± 0.16	4.01 ± 0.10	<LoD	<LoD	<LoD	<LoD	<LoD	<LoD	<LoD	<LoD	2.78	16.69
	**SW5**	0.86 ± 0.12	1.60 ± 0.34	0.97 ± 0.43	<LoD	<LoD	<LoD	<LoD	<LoD	<LoD	<LoD	<LoD	<LoD	3.08	8.60
	**SW6**	1.07 ± 0.43	2.51 ± 0.25	0.66 ± 0.21	3.38	<LoD	<LoD	<LoD	<LoD	<LoD	<LoD	<LoD	<LoD	2.90	18.16
**March-23**	**SW1**	3.60 ± 0.04	3.18*	3.67 ± 0.20	5.83 ± 0.22	<LoD	<LoD	<LoD	<LoD	<LoD	4.15	<LoD	<LoD	10.86	2.28
	**SW2**	3.03 ± 0.00	<LoD	2.69 ± 0.11	4.66 ± 0.04	<LoD	<LoD	<LoD	<LoD	<LoD	3.89	<LoD	<LoD	9.76	3.15
	**SW3**	1.07 ± 0.05	3.18*	3.00 ± 0.23	4.33 ± 0.02	<LoD	<LoD	<LoD	<LoD	<LoD	<LoD	5.27 ± 0.10	<LoD	2.40	3.11
	**SW4**	2.88 ± 0.04	<LoD	4.25 ± 0.04	4.99 ± 0.10	<LoD	<LoD	<LoD	<LoD	<LoD	<LoD	<LoD	<LoD	3.22	1.52
	**SW5**	<LoD	<LoD	1.32 ± 0.37	<LoD	<LoD	<LoD	<LoD	<LoD	<LoD	<LoD	<LoD	<LoD	1.48	1.53
	**SW6**	0.74 ± 0.00	2.93*	<LoD	<LoD	<LoD	<LoD	<LoD	<LoD	<LoD	<LoD	<LoD	<LoD	3.65	1.38

Suppl. Table S14 Levels of faecal indicators, enteric viruses, respiratory viruses in sediments of Albufera Natural Park. Abbreviations: PMMoV, pepper mild mottle virus; HuNoV GI, human norovirus genogroup I; HuNoV GII, human norovirus genogroup II; HAstV, human astrovirus; RV, rotavirus A; HEV, hepatitis E virus; SARS-CoV-2, severe acute respiratory syndrome coronavirus 2; IAV, influenza A virus; RSV, respiratory syncytial virus; pfu, plaque forming units; cfu, colony forming units; gc, genome copies. *one replicate analysed in this sampling campaign. [a]Expressed in (Log10 pfu/g); [b]Expressed in (Log10 cfu/g); [c]Expressed in (Log10 gc/g). Na, not analysed

		Faecal indicators				Enteric viruses (Log_{10} gc/g)					Respiratory viruses (Log_{10} gc/g)		
		**Somatic coliphages[a]**	**Total *E. coli*[b]**	**PMMoV[c]**	**crAssphage[c]**	**HuNoV GI**	**HuNoV GII**	**HAstV**	**RV**	**HEV**	**SARS-CoV-2**	**IAV**	**RSV**
**January-22**	**SD1**	<LoD	<LoD	3.09 ± 0.16	4.60 ± 0.12	<LoD	<LoD	<LoD	<LoD	<LoD	<LoD	<LoD	<LoD
	**SD2**	<LoD	<LoD	2.23 ± 0.18	4.12	<LoD	<LoD	<LoD	<LoD	<LoD	<LoD	<LoD	<LoD
	**SD3**	<LoD	<LoD	4.53 ± 0.02	5.05 ± 0.10	<LoD	<LoD	<LoD	<LoD	4.37	<LoD	<LoD	<LoD
	**SD4**	<LoD	<LoD	2.23 ± 0.45	<LoD	<LoD	<LoD	<LoD	<LoD	<LoD	<LoD	<LoD	<LoD
	**SD5**	<LoD	<LoD	3.16 ± 0.03	<LoD	<LoD	<LoD	<LoD	<LoD	3.46	<LoD	6.23 ± 0.14	<LoD
	**SD6**	<LoD	<LoD	2.71 ± 0.23	3.81	<LoD	<LoD	<LoD	<LoD	4.48 ± 0.34	<LoD	<LoD	<LoD
**July-22**	**SD1**	4.05 ± 0.21	<LoD	3.73 ± 0.00	5.70 ± 0.09	5.35	6.19 ± 0.09	<LoD	<LoD	<LoD	<LoD	<LoD	<LoD
	**SD2**	<LoD	<LoD	2.03 ± 0.23	3.91	<LoD	<LoD	<LoD	<LoD	<LoD	<LoD	<LoD	<LoD
	**SD3**	<LoD	<LoD	1.53	<LoD	<LoD	<LoD	<LoD	<LoD	<LoD	<LoD	<LoD	<LoD
	**SD4**	<LoD	<LoD	3.06 ± 0.04	4.58 ± 0.06	<LoD	<LoD	5.69 ± 0.00	<LoD	<LoD	<LoD	<LoD	<LoD
	**SD5**	<LoD	<LoD	1.48 ± 0.04	<LoD	5.10	<LoD	4.91	<LoD	<LoD	<LoD	<LoD	<LoD
	**SD6**	<LoD	<LoD	1.60	4.43 ± 0.40	5.39 ± 0.01	<LoD	5.05 ± 0.05	<LoD	<LoD	<LoD	<LoD	<LoD
**March-23**	**SD1**	Na				Na					Na		
	**SD2**	<LoD	<LoD	2.29 ± 0.05	4.51 ± 0.14	4.95	6.11	<LoD	<LoD	<LoD	<LoD	<LoD	<LoD
	**SD3**	<LoD	<LoD	3.37 ± 0.06	4.62 ± 0.59	<LoD	<LoD	<LoD	<LoD	<LoD	<LoD	<LoD	<LoD
	**SD4**	<LoD	<LoD	<LoD	5.18 ± 0.10	<LoD	<LoD	<LoD	7.02 ± 0.02	<LoD	<LoD	<LoD	<LoD
	**SD5**	<LoD	<LoD	2.05 ± 0.23	4.47	5.37	<LoD	<LoD	<LoD	<LoD	<LoD	<LoD	<LoD
	**SD6**	<LoD	<LoD	1.58 ± 0.36	<LoD	4.75	<LoD	<LoD	<LoD	<LoD	<LoD	<LoD	<LoD

Suppl. Table S15 Levels of faecal indicators, enteric viruses, respiratory viruses and mean recoveries of PEDV and MgV in surface waters of Ebro River region. Abbreviations: PMMoV, pepper mild mottle virus; HuNoV GI, human norovirus genogroup I; HuNoV GII, human norovirus genogroup II; HAstV, human astrovirus; RV, rotavirus A; HEV, hepatitis E virus; SARS-CoV-2, severe acute respiratory syndrome coronavirus 2; IAV, influenza A virus; RSV, respiratory syncytial virus; PEDV, porcine epidemic diarrhoea virus; MgV, mengovirus; pfu, plaque forming units; cfu, colony forming units; gc, genome copies. *one replicate analysed in this sampling campaign. [a]Expressed in (Log10 pfu/L); [b]Expressed in (Log10 cfu/L); [c]Expressed in (Log10 gc/L)

		**Faecal indicators**				**Enteric viruses (Log_{10} gc/L)**					**Respiratory viruses (Log_{10} gc/L)**			**Process control viruses (% recovery)**	
		**Somatic coliphages[a]**	**Total *E. coli*[b]**	**PMMoV[c]**	**crAssphage[c]**	**HuNoV GI**	**HuNoV GII**	**HAstV**	**RV**	**HEV**	**SARS-CoV-2**	**IAV**	**RSV**	**PEDV**	**MgV**
**January-22**	**SW7**	2.32 ± 0.06	<LoD	4.30 ± 0.20	4.32 ± 0.55	<LoD	5.66	4.17	5.35 ± 0.01	3.12	<LoD	<LoD	<LoD	14.72	16.68
	**SW8**	2.85 ± 0.00	<LoD	3.90 ± 0.05	4.45 ± 1.03	<LoD	5.53 ± 0.15	<LoD	5.52 ± 0.06	3.36	<LoD	<LoD	<LoD	6.20	8.41
	**SW9**	2.29 ± 0.07	<LoD	3.78 ± 0.04	3.49 ± 0.71	<LoD	5.38	4.64 ± 0.01	<LoD	4.03 ± 0.33	<LoD	<LoD	<LoD	11.52	10.48
	**SW10**	1.93 ± 0.13	2.24*	3.43 ± 0.02	3.86 ± 0.75	<LoD	5.50	3.70	<LoD	3.31	<LoD	<LoD	<LoD	3.92	8.41
	**SW11**	2.01 ± 0.05	<LoD	3.70 ± 0.03	3.83 ± 0.00	<LoD	5.44	<LoD	<LoD	3.51	<LoD	<LoD	<LoD	8.51	14.09
**July-22**	**SW7**	3.47 ± 0.04	2.06*	3.43 ± 0.14	4.28 ± 0.12	<LoD	5.33	<LoD	5.76 ± 0.18	<LoD	3.80 ± 0.09	<LoD	<LoD	2.49	3.31
	**SW8**	3.99 ± 0.09	4.10*	3.74 ± 0.13	4.83 ± 0.09	<LoD	5.48	6.00 ± 0.31	<LoD	<LoD	<LoD	<LoD	<LoD	8.08	2.28
	**SW9**	1.16 ± 0.30	2.28*	2.94 ± 0.06	<LoD	<LoD	<LoD	<LoD	<LoD	<LoD	3.65	<LoD	<LoD	11.14	1.02
	**SW10**	3.88 ± 0.01	1.18*	2.25 ± 0.06	4.37 ± 0.06	<LoD	<LoD	<LoD	<LoD	<LoD	<LoD	<LoD	<LoD	1.90	1.10
	**SW11**	3.44 ± 0.03	1.88*	2.21 ± 0.04	3.69 ± 0.54	<LoD	<LoD	<LoD	<LoD	<LoD	<LoD	<LoD	<LoD	5.69	2.10
**March-23**	**SW7**	2.88 ± 0.09	<LoD	3.78 ± 0.51	3.66	<LoD	<LoD	<LoD	<LoD	<LoD	<LoD	<LoD	<LoD	5.84	2.55
	**SW8**	3.72 ± 0.01	<LoD	3.90 ± 0.08	4.66 ± 0.08	<LoD	5.17	<LoD	6.18	<LoD	<LoD	<LoD	<LoD	10.79	4.80
	**SW9**	1.21 ± 0.07	<LoD	3.52 ± 0.21	4.48 ± 0.21	<LoD	5.06	<LoD	<LoD	<LoD	<LoD	<LoD	<LoD	3.78	2.02
	**SW10**	2.37 ± 0.28	2.59*	2.49 ± 0.13	4.22 ± 0.21	4.25	<LoD	<LoD	<LoD	<LoD	<LoD	<LoD	<LoD	4.38	1.93
	**SW11**	1.42 ± 0.06	<LoD	2.51 ± 0.15	3.15 ± 0.05	4.04	<LoD	<LoD	<LoD	<LoD	<LoD	<LoD	<LoD	9.94	2.59

Suppl. Table S16 Levels of faecal indicators, enteric viruses, respiratory viruses in sediments of Ebro River region. Abbreviations: PMMoV, pepper mild mottle virus; HuNoV GI, human norovirus genogroup I; HuNoV GII, human norovirus genogroup II; HAstV, human astrovirus; RV, rotavirus A; HEV, hepatitis E virus; SARS-CoV-2, severe acute respiratory syndrome coronavirus 2; IAV, influenza A virus; RSV, respiratory syncytial virus; pfu, plaque forming units; cfu, colony forming units; gc, genome copies. *one replicate analysed in this sampling campaign. [a]Expressed in (Log10 pfu/g); [b]Expressed in (Log10 cfu/g); [c]Expressed in (Log10 gc/g)

		**Faecal indicators**				**Enteric viruses (Log_{10} gc/g)**					**Respiratory viruses (Log_{10} gc/g)**		
		**Somatic coliphages[a]**	**Total *E. coli*[b]**	**PMMoV[c]**	**crAssphage[c]**	**HuNoV GI**	**HuNoV GII**	**HAstV**	**RV**	**HEV**	**SARS-CoV-2**	**IAV**	**RSV**
**January-22**	**SD7**	<LoD	<LoD	<LoD	<LoD	<LoD	<LoD	<LoD	<LoD	3.68	<LoD	<LoD	<LoD
	**SD8**	<LoD	<LoD	2.97	<LoD	<LoD	<LoD	<LoD	<LoD	<LoD	<LoD	<LoD	<LoD
	**SD9**	<LoD	<LoD	2.93 ± 0.13	3.96 ± 0.08	<LoD	<LoD	<LoD	<LoD	<LoD	<LoD	<LoD	<LoD
	**SD10**	<LoD	<LoD	2.98 ± 0.07	4.38 ± 0.03	<LoD	<LoD	5.11 ± 0.11	<LoD	<LoD	<LoD	<LoD	<LoD
**July-22**	**SD7**	4.29 ± 0.04	4.35 ± 0.25	4.60 ± 0.08	<LoD	5.12	5.94	6.71 ± 0.01	<LoD	<LoD	<LoD	<LoD	<LoD
	**SD8**	<LoD	<LoD	2.52 ± 0.03	<LoD	<LoD	<LoD	<LoD	<LoD	<LoD	<LoD	<LoD	<LoD
	**SD9**	<LoD	<LoD	1.87	3.84 ± 0.17	5.35	<LoD	7.13	<LoD	<LoD	<LoD	<LoD	<LoD
	**SD10**	<LoD	<LoD	<LoD	<LoD	4.97	<LoD	<LoD	<LoD	<LoD	<LoD	<LoD	<LoD
**March-23**	**SD7**	<LoD	<LoD	4.56 ± 0.02	7.42 ± 0.08	<LoD	7.06 ± 0.06	<LoD	6.65	<LoD	<LoD	<LoD	<LoD
	**SD8**	<LoD	3.00	1.28	6.02 ± 0.04	5.15 ± 0.05	<LoD	<LoD	<LoD	<LoD	<LoD	<LoD	<LoD
	**SD9**	2.00	<LoD	2.07 ± 0.07	<LoD	4.76 ± 0.08	<LoD	<LoD	<LoD	<LoD	<LoD	<LoD	<LoD
	**SD10**	<LoD	<LoD	2.36	4.33 ± 0.10	<LoD	<LoD	<LoD	<LoD	<LoD	<LoD	<LoD	<LoD
